# TSPYL5-driven G3BP1 nuclear membrane translocation facilitates p53 cytoplasm sequestration via accelerating RanBP2-mediated p53 sumoylation and nuclear export in neuroblastoma

**DOI:** 10.1038/s41419-025-07694-x

**Published:** 2025-05-03

**Authors:** Zhaokun Wang, Yunqiang Liu, Yangwei Zhang, Jiaying Shi, Shengyu Xie, Ming Yi, Xinyue Zhang, Dachang Tao, Yuan Yang

**Affiliations:** Department of Medical Genetics, State Key Laboratory of Biotherapy, West China Hospital, Sichuan University, Chengdu, China

**Keywords:** Paediatric cancer, Predictive markers

## Abstract

Cytoplasmic sequestration of wild-type p53, representing a nonmutational event of p53 activity suppression, is a characteristic phenotype of undifferentiated neuroblastoma (NB); however, the underlying mechanism is yet to be defined. In the present study, we observed that TSPYL5 effectively tethers p53 in the cytoplasm and greatly inhibits its function as a transcription factor. Mechanistically, the binding of TSPYL5 with G3BP1 enhances G3BP1 Ser149 phosphorylation to drive G3BP1 nuclear membrane translocation, which recruits more p53 for nucleoporin RanBP2 by the formation of the RanBP2-G3BP1-p53 complex. Thus, the accelerating p53 sumoylation promotes its nuclear export. With this signal pathway, TSPYL5 augments the malignant characteristics of neuroblastoma cells. Our findings unravel a detailed TSPYL5-driven molecular axis that sheds light on the regulating system of the p53 sumoylation-based cytoplasmic sequestration in NB cells, paving the way for the novel therapeutic opportunities for NB cancers by antagonizing TSPYL5 function.

## Introduction

Neuroblastoma (NB), the most common cause of cancer-related death in childhood, is responsible for ~15% of pediatric cancer deaths [1]. NB commonly originates from the undifferentiated neural crest cells during fetal development and about 65% of NB specimens were examined to possess the characteristics of cancer stem cells (CSCs) such as chemoradiotherapy resistance and distant metastasis [2]. It is well known that the tumor suppressor p53 plays crucial roles in the cellular processes including self-renewal, differentiation, and reprogramming of stem cells [3], and loss of p53 has been demonstrated to account for CSCs amplification in some tumors [4, 5]. p53 is rarely mutated in neuroblastoma, however, the cytoplasmic sequestration of wild-type p53, which is proposed as a non-mutational mechanism for p53 inactivation, is present in about 95% of undifferentiated NB cases [6]. Thus, it is greatly possible that the p53 cytoplasmic sequestration dramatically reduces p53 transcriptional activity and then increases the CSC amount of NB, contributing to poor prognosis of NB patients. Indeed, reactivating wild-type p53 has been suggested as a promising strategy to improve NB therapy [7]. Before that, however, the mechanism underlying the cytoplasmic p53 sequestration needs to be intensively examined in NB.

Several mechanisms have been proposed for cytoplasmic p53 sequestration. It is frequently observed that the C-terminal domain of p53 is anchored by other proteins or long noncoding RNAs [8, 9], forming a large protein aggregate to tether p53 in the cytoplasm. Another more common and efficient factor responsible for p53 cytoplasm localization is the ubiquitylation status in its C-terminus, in which monoubiquitination facilitates nuclear export [10], and recent report further suggests that the SUMO modification of monoubiquitinated p53 promotes its cytoplasmic translocation [11]. However, the regulators driving and controlling the p53 posttranslational modification (PTM)-mediated cytoplasmic accumulation are still not well understood.

TSPY-like 5 (TSPYL5), an autosome-encoded homologous protein of testis-specific protein Y-linked 1 (TSPY1), is variably expressed in human tissues and mainly in the testis. Additionally, TSPYL5 has also been reported to be abundantly expressed in many adult tumors, correlated with their poor progression [12–14]. As a conserved C-terminal nucleosome assembly protein, TSPYL5 is expected to play roles in chromosome assembly, histone replacement, tissue-specific gene transcriptional regulation, and cell cycle regulation. Notably, TSPYL5 interacts with USP7 (ubiquitin specific peptidase 7) to reduce the tumor suppressor activity of p53, overriding p53-dependent proliferation arrest [12], promoting endothelial cell proliferation and angiogenesis [15], and protecting POT1 (protection of telomeres 1) from proteasomal degradation in ALT (alternative lengthening of telomeres)-positive cells [16]. Additionally, TSPYL5 has been demonstrated to be critical for maintaining CSC-like characteristics of cancer cells [13]. Importantly, our recent investigation suggested that the high expression of TSPYL5 effects the cytoplasmic p53 sequestration and transcriptional inactivity in NB cells, providing an opportunity to shed light on the mechanism underlying the cytoplasmic sequestration of p53 by exploring the role of TSPYL5 in regulating p53 nucleocytoplasmic transport in NB tumors.

In this study, we unveils the regulation mechanism underlying the cytoplasmic p53 sequestration of NB cells, by which TSPYL5 drives G3BP1 (G3BP stress granule assembly factor 1)/RanBP2 (RAN binding protein 2)/SUMO (small ubiquitin-related modifier)-mediated p53 sumoylation, enhancing p53 nuclear export signal and inhibiting its tumor suppressor activity.

## Results

### TSPYL5 augments the malignant characteristics of NB cells by suppressing the transcriptional activity of p53

**Fig. 1:**
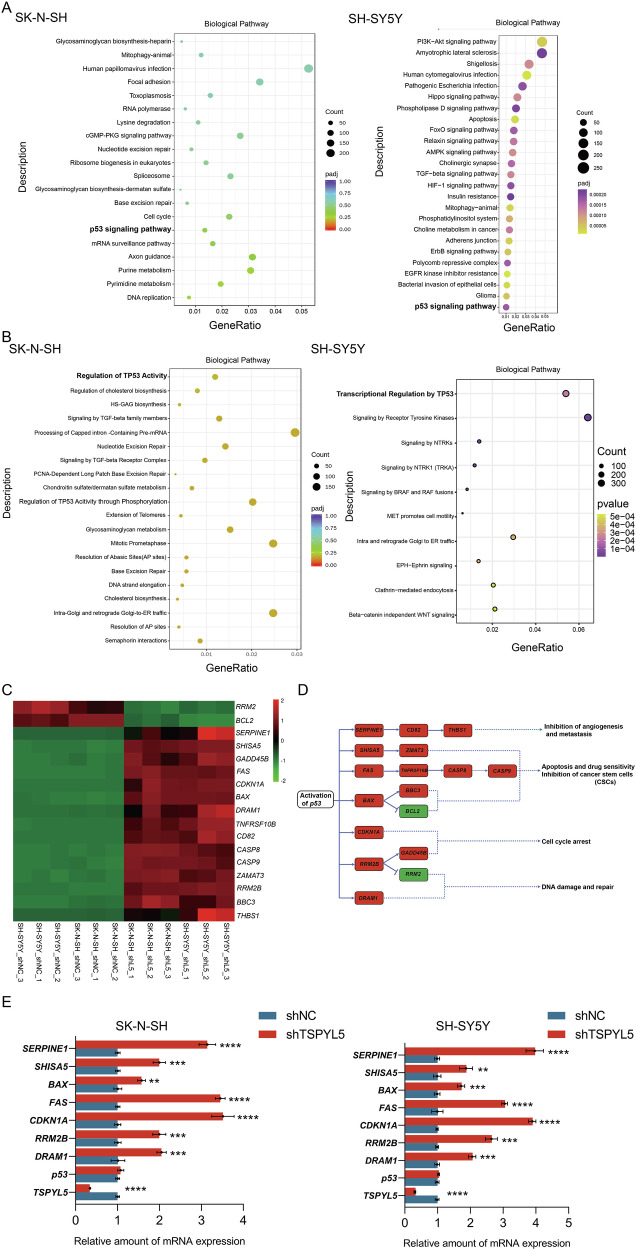
TSPYL5 inhibits p53-mediated transcriptional activity. **A** KEGG enrichment analysis of the differentially expressional genes (DEGs) observed in the RNA-seq of SK-N-SH and SH-SY5Y cells transfected with shNC and shTSPYL5. **B** Reactome enrichment analysis of DEGs of the TSPYL5-knocked down SK-N-SH and SH-SY5Y cells. **C** Heatmap of the 17 DEGs involved in the p53 signaling pathway in the SK-N-SH and SH-SY5Y cells. The unit for the color scale was the z score of log_2_ expression data shown on the right side of the diagram. **D** The location of the p53-related DEGs in the p53 signaling pathway as annotated by KEGG. The names of upregulated DEGs in the TSPYL5-knocked down cells were covered in red and those of downregulated DEGs were covered in green. The functions of the DEGs were shown in the right side of the diagram. **E** The validation analysis of the expression of seven p53-directly targeted genes in the SK-N-SH and SH-SY5Y transfected with shNC and shTSPYL5. The mean ± SD from three experiments was plotted. *p < 0.05, **p < 0.01, ***p < 0.001.

We firstly found that the high expression of *TSPYL5* in NB tumors and cells (Supplementary Fig. 1a, b), and the single-cell transcriptomes of NB tissues further demonstrated its specific expression in neuroendocrine (NE) cells (Supplementary Fig. 1c–f), the representative malignant cells of NB tumors [17]. To investigate the role of TSPYL5 in NB tumors, we selected two regularly used cell lines in NB studies, including SK-N-SH and SH-SY5Y. After confirming that TSPYL5 was abundantly expressed in the cytoplasm (Supplementary Fig. 2a), we established the TSPYL5-knockdown models of the two cell lines (Supplementary Fig. 2b). Then, we explored the potential function of TSPYL5 by RNA-seq of the two models. As a result, more than 14,000 DEGs were observed in both cell lines with similar gene expression patterns (GSE223866) (Supplementary Fig. 3a, b). Even if the filtering threshold was been set to |log2fold change| > 2 and Padj < 0.05, 5763 and 5880 DEGs were identified in SK-N-SH and SH-SY5Y, respectively. Interestingly, the p53 signaling pathway was enriched via both KEGG and Reactome analysis of SK-N-SH DEGs and the similar result obtained also in RNA-seq analysis of SH-SY5Y cells (Fig. 1A, B). Several cell functions associated with the p53 signaling pathway, including DNA replication, RNA splicing, and intercellular adhesion [18–20], were also enriched via GO analysis (Supplementary Fig. 3c).

It is well known that p53 functions as a transcription factor to suppress the malignant transformation of cells [21]. For this, our transcriptome data were further excavated to determine the DEGs involved in the p53 signaling pathway. As a result, a total of 17 p53-related genes were confirmed to be remarkably differentially expressed in both NB cell lines (|log_2_fold change| > 2, *P*adj < 0.05) (Fig. 1C), totally suggesting the upregulation of p53 function in TSPYL5-knocked down NB cells (Fig. 1D). Then, we extracted the data from TARGET (https://ocg.cancer.gov/programs/target) to examine the correlation between the expression of *TSPYL5* and these p53-targeted DEGs in NB tissues. The results showed that the correlations between the expression of *TSPYL5* and ten p53-targeted genes, including *SERPINE1*, *THBS1*, *FAS*, *CD82*, *CASP8*, *CASP9*, *BBC3*, *DRAM1 SHISA5*, and *GADD45B*, were consistent with those from our transcriptomic analysis (Supplementary Fig. 4a–j). For each of seven p53-directly targeted genes, including *SERPINE1*, *SHISA5*, *BAX*, *FAS*, *CDKN1A*, *RRM2* and *DRAM1*, the significantly upregulated expression in both TSPYL5-knockdown SK-N-SH and SH-SY5Y cells was further confirmed (Fig.1E). In addition, reanalysis of the single-cell transcriptome further demonstrated a negative regulation of TSPYL5 on the p53 signaling pathway (Supplementary Fig. 3d). These findings suggest that TSPYL5 effectively suppresses p53 transcriptional activity in NB cells.

**Fig. 2:**
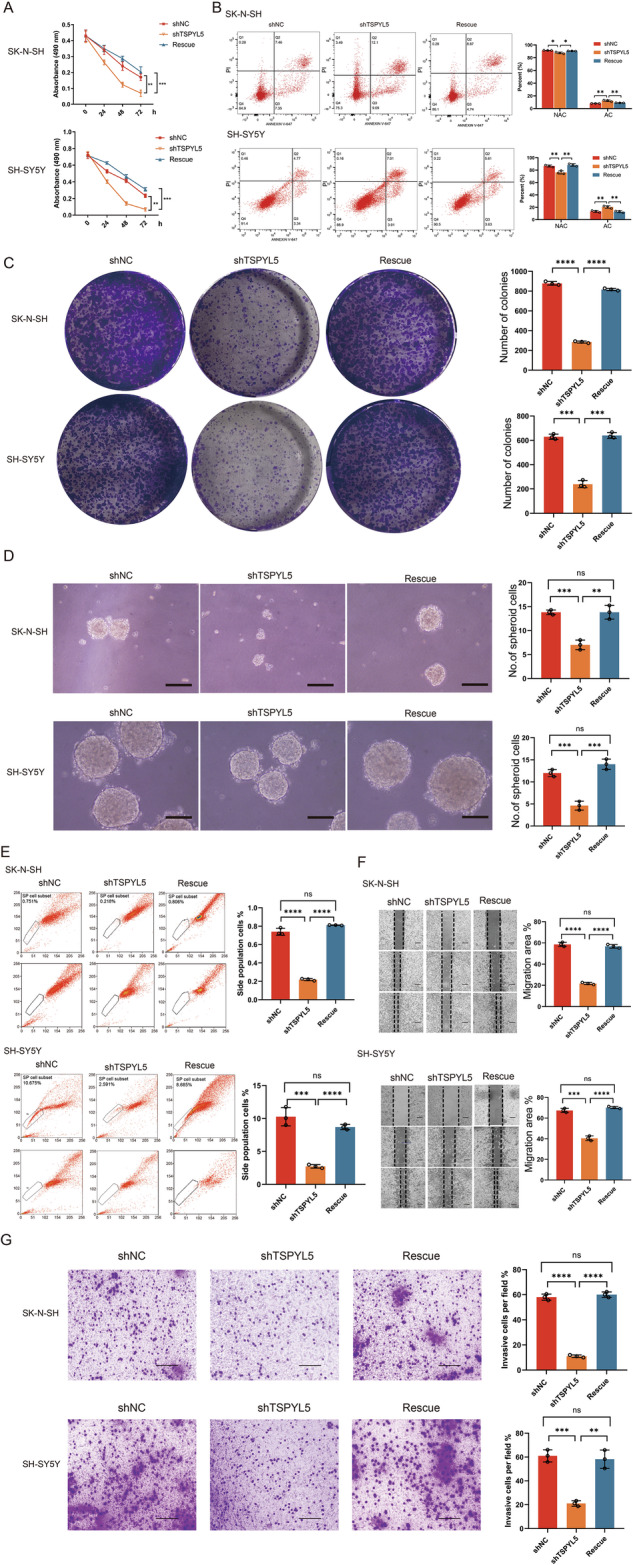
TSPYL5 augments the malignant characteristics of NB cells. **A** CCK-8 assays showing the sensitivity to cisplatin in the SK-N-SH and SH-SY5Y cells transfected with shNC or shTSPYL5. **B** Flow cytometry assays showing the apoptosis induced by cisplatin in the SK-N-SH and SH-SY5Y cells transfected with shNC and shTSPYL5. **C** Colony formation assays in the SK-N-SH and SH-SY5Y cells transfected with shNC or shTSPYL5. **D** Sphere formation assays in the SK-N-SH and SH-SY5Y cells transfected with shNC or shTSPYL5. Scale bar = 25 μm. **E** Side population ratio assays in the SK-N-SH and SH-SY5Y cells transfected with shNC and shTSPYL5. **F**, **G** Migration and invasion assays in the SK-N-SH and SH-SY5Y cells transfected with shNC and shTSPYL5. Scale bar = 50 μm. Rescue assays was used to exclude the off-target effect. ns = no significant difference. The mean ± SD from three experiments was plotted. *p < 0.05, **p < 0.01, ***p < 0.001.

Among these p53-related DEGs, *SERPINE1*, *CD82* and *THBS1* inhibit metastasis, invasion and angiogenesis of tumor cells [22], *SHISA5*, *ZMAT3*, *FAS*, *TNFRSF10B*, *CASP8*, *CASP9*, *BAX*, *BBC3*, and *BCL2* promote cell apoptosis and increase sensitivity to drug [23], *FAS* and *TNFRSF10B* have also been reported to support CSC maintenance [24], *CDKN1A*, *RRM2*, *GADD45B*, and *RRM2B* mediate cell cycle arrest [25], and *DRAM1*, *RRM2*, and *RRM2B* are also required for the repair of DNA damage [26]. To investigate whether the suppression of p53 activity by TSPYL5 observably affects the functions of NB cells, we examined the p53-related cell phenotypes, including cell proliferation, apoptosis, colonal and sphere formation, side population ratio, invasiveness, and migration of NB cells with different levels of TSPYL5. The results showed that the decrease in TSPYL5 seriously reduced the viability and increased the apoptosis of NB cells treated with cisplatin, a commonly used chemotherapeutic agent, and the rescue of *TSPYL5* expression restored the proliferation and inhibited the apoptosis of cisplatin-treated NB cells (Fig. 2A, B). Additionally, the knockdown of TSPYL5 significantly reduced the number of cell colonies and the size of sphere as well as the ratio of side population cells (Fig. 2C–E), clearly inhibited the invasiveness and migration of NB cells (Fig. 2F, G) and while the exogenous expression of TSPYL5 rescued the above phenotypes (Fig. 2C–G). These observations suggest that the knockdown of TSPYL5 expression contributes to the inhibition of NB malignant progression via activating the p53 tumor suppressor.

In order to investigate whether TSPYL5’s impacts on NB malignant progression are dependent on p53 activity, we reduced the p53 expression in the TSPYL5-knockdown NB cells using the *TP53*-targeted SiRNA (Supplementary Fig. 5a, b). We found that the downregulated p53 expression significantly decreased its targeted genes expression which had been augmented in the TPSYL5-knockdown NB cells (Supplementary Fig. 5c). Furthermore, we observed that the p53 decreasing enhanced the viability and reduced the apoptosis of the TPSYL5-knockdown NB cells treated with cisplatin (Supplementary Fig. 6a, b). Additionally, further reducing the p53 expression in the TPSYL5-knockdown NB cells obviously increased the number of cell colonies and the size of sphere as well as the ratio of side population cells (Supplementary Fig. 6c–e), and also enhanced the invasiveness and migration capacity (Supplementary Fig. 6f, g). Taken together, these results further supported that TSPYL5 augments the malignant characteristics of NB cells through regulating the p53 signaling pathway.

### TSPYL5 effects cytoplasmic p53 sequestration by promoting SUMO-mediated nuclear export

**Fig. 3:**
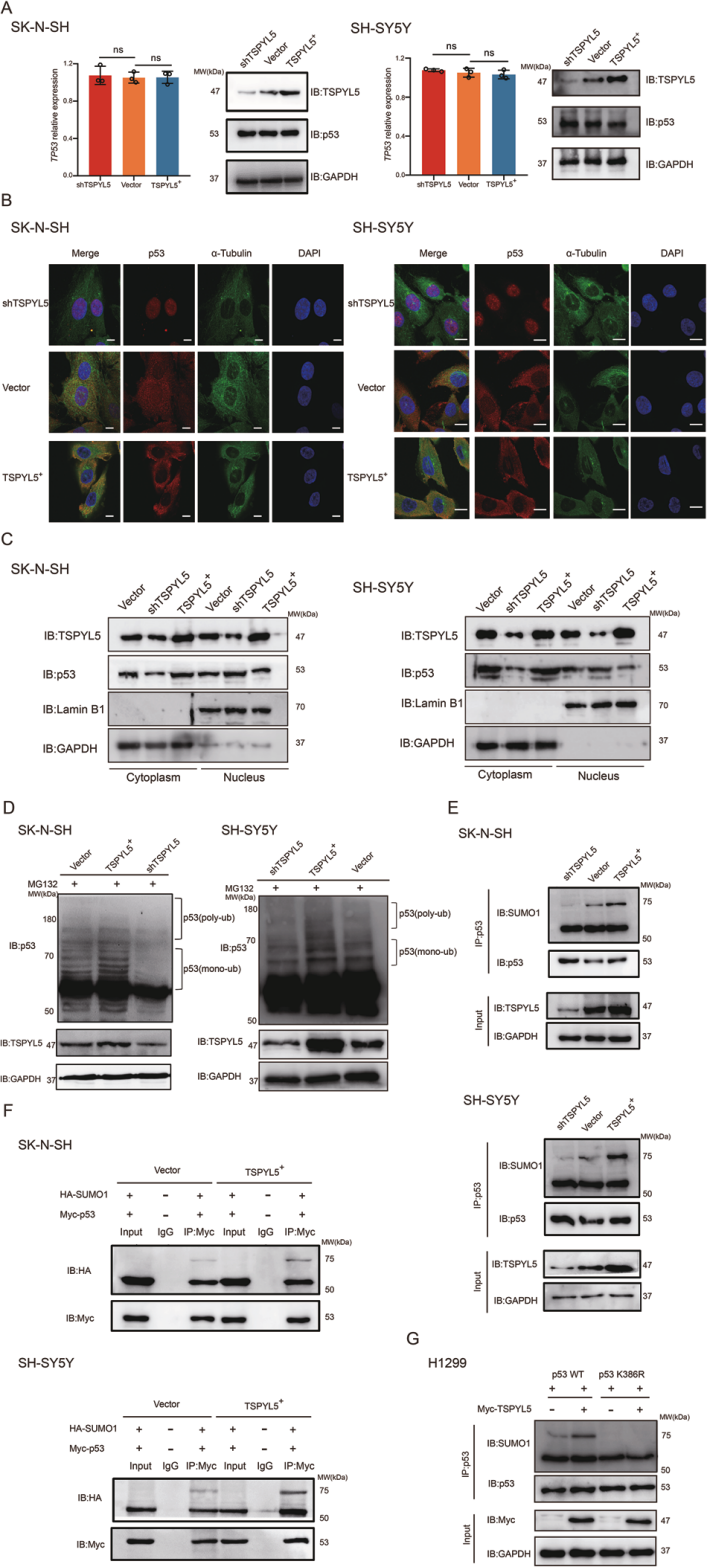
TSPYL5 enhances p53 cytoplasm sequestration by promoting p53 sumoylation. **A** The comparison of p53 expression in the SK-N-SH and SH-SY5Y with different TSPYL5 expression level. ns no significant difference. **B** The comparison of p53 subcellular localization in the SK-N-SH and SH-SY5Y with different TSPYL5 expression level by immunofluorescence analysis. Cell nuclei were counterstained with 4,6-diamidino-2-phenylindole (DAPI). Scale bar = 10 μm. **C** The comparison of p53 subcellular localization in the SK-N-SH and SH-SY5Y with different TSPYL5 expression level by the nucleus-plasm separation experiment. **D** The ubiquitination pattern of p53 in the SK-N-SH and SH-SY5Y cells with different TSPYL5 expression level. **E** The comparison of the level of SUMO1 immunoprecipitated with p53 in the SK-N-SH and SH-SY5Y cells with different TSPYL5 expression level. **F** The comparison of the level of exogenous HA-SUMO1 immunoprecipitated with Myc-p53 in the SK-N-SH and SH-SY5Y with different TSPYL5 expression level. **G** The comparison of the level of SUMO1 immunoprecipitated with exogenous Flag-p53 (wild-type) and Flag-p53 (K386R) in the H1299 with and without exogenous Myc-TSPYL5 expression.

In NB cells, the inhibition of p53 function by TSPYL5 was not due to a decrease in the mRNA and protein levels of p53 (Fig. 3A). In this case, our first idea was whether TSPYL5 could regulate p53 subcellular localization. Indeed, previous studies have reported the cytoplasmic sequestration of wild-type p53 in undifferentiated NB tumors [27]. In the present study, we found that p53 was mainly located in the cytoplasm of SK-N-SH and SH-SY5Y cells, while it was redistributed in the nucleus of TSPYL5-knockdown cells, and the rescue of TSPYL5 expression restored cytoplasmic p53 localization (Fig. 3B). This observation was confirmed by the nucleus-plasm separation experiment (Fig. 3C, Supplementary Fig. 7a, b). These results suggest that TSPYL5 suppresses the transcriptional activity of p53 by facilitating cytoplasmic p53 sequestration in NB cells.

The PTMs of p53 have been suggested to determine the subcellular localization of p53 [10, 11]. In this case, we compared the degree of p53 ubiquitination among NB cells with different TSPYL5 expression levels. Impressively, we observed a high level of monoubiquitinated, not polyubiquitinated, p53 in NB cells and found that the monoubiquitination of p53 was enhanced with increasing TSPYL5 expression (Fig. 3D). Previous studies have demonstrated that high levels of MDM2 (MDM2 proto-oncogene) are commonly required for p53 polyubiquitination and degradation, while low levels of MDM2 help the attachment of a single ubiquitin to the C-terminal lysines of p53 to initiate other modifications of monoubiquitinated p53 and nuclear export [10, 28]. Indeed, we observed that SK-N-SH and SH-SY5Y cells presented a lower level of MDM2 than other cells reported to possess a high level of MDM2 expression (Supplementary Fig. 8a) and that TSPYL5 could not obviously change the status of low MDM2 (Supplementary Fig. 8b). All of these results imply that under the unique molecular environment of low MDM2 levels, the high level of monoubiquitinated p53 in NB cells overexpressing TSPYL5 prepares for its nuclear export.

Monoubiquitinated p53 can be sumoylated, and p53 sumoylation can accelerate its nuclear export and cytoplasmic localization [10, 29]. It is reasonable, therefore, to presume that a considerable amount of cytoplasmic p53 may be SUMO-modified p53 in NB cells. To test this hypothesis, we performed IP experiments of sumoylated p53 in both SK-N-SH and SH-SY5Y cells, and the results clearly showed an increase in sumoylated p53 when TSPYL5 was overexpressed (Fig. 3E). Then, using HA-tagged SUMO1 and Myc-tagged p53, we confirmed an enhanced interaction between SUMO1 and p53 in cells overexpressing TSPYL5 (Fig. 3F). Furthermore, we constructed a vector expressing p53 harboring K386R, a missense mutation inhibiting p53 sumoylation [30]. In H1299 cells lacking endogenous p53, we observed an upregulated sumoylation level of exogenous wild-type p53 when overexpressing TSPYL5, while such a role was not found in H1299 cells expressing exogenous p53-K386R (Fig. 3G), further supporting the promotion of p53 sumoylation by TSPYL5. All of these results suggest that TSPYL5 expression facilitates cytoplasmic p53 sequestration by enhancing p53 sumoylation-mediated nuclear export in NB cells. Additionally, a previous study reported the contribution of aberrant p53 hyperubiquitylation to cytoplasmic p53 sequestration in NB [31]. Actually, the ubiquitylated p53 in the report is almost monoubiquitiylated. Overall, we speculate that the extremely low level of polyubiquitination prolongs the half-life of wild-type p53 so that a great amount of monoubiquitinated/sumoylated p53 accumulates in the cytoplasm of NB cells.

### TSPYL5-mediated p53 sumoylation depends on its interaction with G3BP1

**Fig. 4:**
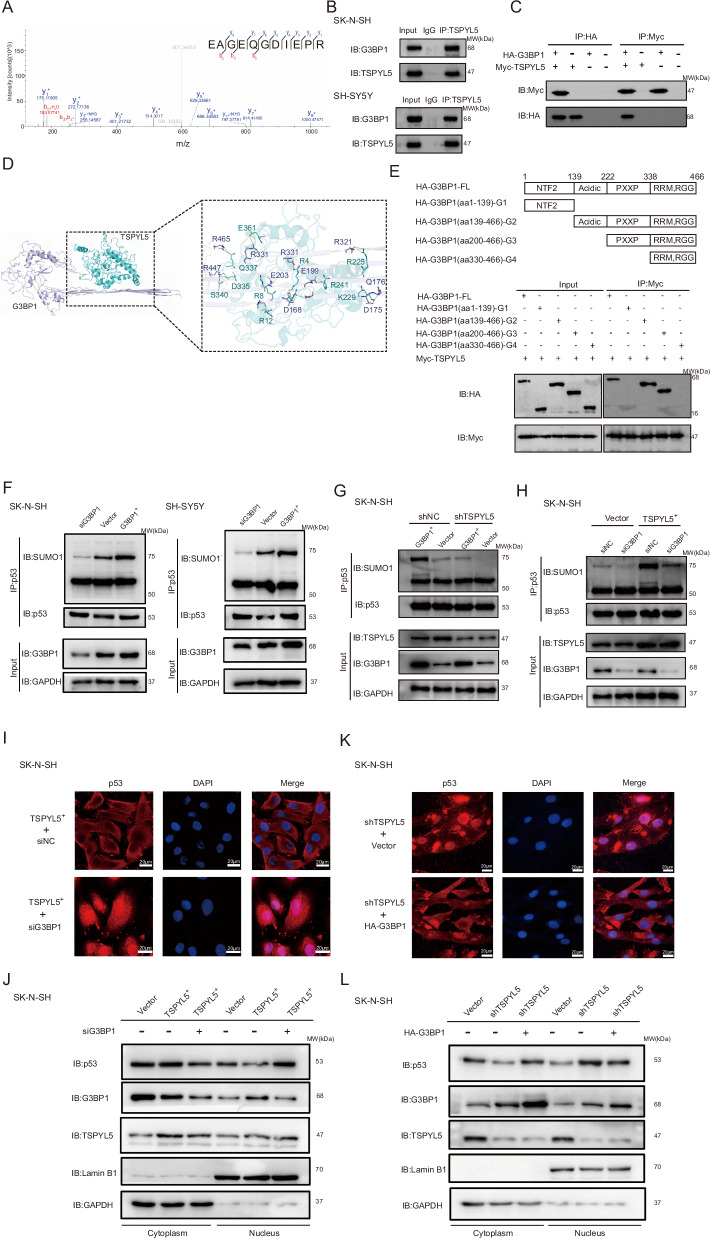
TSPYL5-promoted p53 sumoylation depends on its interaction with G3BP1. **A** Mass spectrometry analysis identified G3BP1 in the binding protein pool of TSPYL5. **B** Endogenous interaction between TSPYL5 and G3BP1 was determined using Co-IP with anti-TSPL5 or anti-G3BP1 antibodies in SK-N-SH and SH-SY5Y cells. **C** Exogenous interaction between TSPYL5 and G3BP1 was determined using Co-IP with anti-HA or anti-MYC antibodies in SK-N-SH cells. **D** Interaction between the PXXP domain of G3BP1 and TSPYL5 was determined using Co-IP with anti-HA or anti-MYC antibodies. **E** The binding sites of TSPYL5 and G3BP1 proposed by computational docking model. TSPYL5 molecule was shown as blue sticks, and G3BP1 was shown as purple sticks. **F** G3BP1 promoted p53 sumoylation in SK-N-SH and SH-SY5Y cells. **G** The overexpression of G3BP1 increased the sumoylation level of p53 in SK-N-SH cells with TSPYL5 knockdown. **H** The knockdown of G3BP1 inhibited the positively effect of TSPYL5 on the sumoylation level of p53 in SK-N-SH cells. **I** Immunofluorescence analysis showed the increase of p53 nuclear translocation by the knockdown of G3BP1 in SK-N-SH cells with TSPYL5 overexpression. Scale bar = 20 μm. **J** Nucleus-plasm separation examination showed the increase of p53 nuclear translocation by the knockdown of G3BP1 in SK-N-SH cells with TSPYL5 overexpression. **K** Immunofluorescence analysis showed the increase of p53 cytoplasm localization by the overexpression of G3BP1 in SK-N-SH cells with TSPYL5 knockdown. Scale bar = 20 μm. **L** Nucleus-plasm separation examination assay showed the increase of p53 cytoplasm localization by the overexpression of G3BP1 in SK-N-SH cells with TSPYL5 knockdown.

To explore the mechanism underlying the promotion of p53 sumoylation by TSPYL5, we first investigated the function of the top 50 DEGs obtained from the RNA-seq analysis of the TSPYL5-knockdown cells, but we did not find any gene reported to be involved in p53 sumoylation (Supplementary Tables 4 and 5). Then, the TSPYL5-interacting proteins were investigated in NB cells by Co-IP following LC‒MS/MS analysis. Consequently, G3BP1, a high-scoring partner of TSPYL5 (Fig. 4A), caught our attention since the protein has been suggested to promote cytoplasmic p53 localization [32], although the underlying mechanism was not associated with p53 modification. The following IP and western blotting analysis confirmed the binding between G3BP1 and TSPYL5 (Fig. 4B, C), which was supported by the computational docking model of the two molecules (Fig. 4D). The G3BP1 peptide from 200 aa to 330 aa containing the PXXP domain was further identified to be necessary for its interaction with TSPYL5 (Fig. 4E).

Furthermore, we found that G3BP1 increased the sumoylation of p53 in NB cells (Fig. 4F). Therefore, it is possible that the promotion of TSPYL5 on sumoylation and cytoplasmic localization of p53 is dependent on its interaction with G3BP1. To prove this idea, we performed IP experiments of sumoylated p53 in both SK-N-SH and SH-SY5Y cells with different levels of TSPYL5 and G3BP1. We clearly observed a dependence of TSPYL5-mediated p53 sumoylation on G3BP1 (Fig. 4G, H). Meanwhile, we performed immunofluorescence analysis and nucleocytoplasmic separation examination and observed that the downregulation of G3BP1 in TSPYL5-overexpressing SK-N-SH cells decreased the nuclear export of p53 and that G3BP1 overexpression increased the nuclear export of p53 in TSPYL5-knockdown cells (Fig. 4I–L), indicating that the regulation of p53 cytoplasm translocation by TSPYL5 is dependent on the function of G3BP1.

Considering that G3BP1 acts as the core protein of stress granules (SGs) in cells under stress [33], we tested the involvement of SGs formation in TSPYL5/G3BP1-mediated p53 sumoylation. Treating cells with a frequently used SGs inducer arsenate, we clearly observed a cytoplasmic punctate pattern of G3BP1(Supplementary Fig. 9a) and the increased phosphorylation of EIF2S1 (eukaryotic translation initiation factor 2 alpha at S51) (Supplementary Fig. 9b), a marker for showing SGs formation [34]. However, SGs formation did not interrupt the TSPYL5-increased p53 sumoylation and cytoplasmic location (Supplementary Fig. 9a, c). The interaction between TSPYL5 and G3BP1 were not reduced (Supplementary Fig. 9d), indicating that in cells with SGs, TSPYL5 still interacts with G3BP1 to activate G3BP1-mediated p53 sumoylation and nuclear export.

### TSPYL5/G3BP1-mediated p53 sumoylation requires RanBP2 involvement

**Fig. 5:**
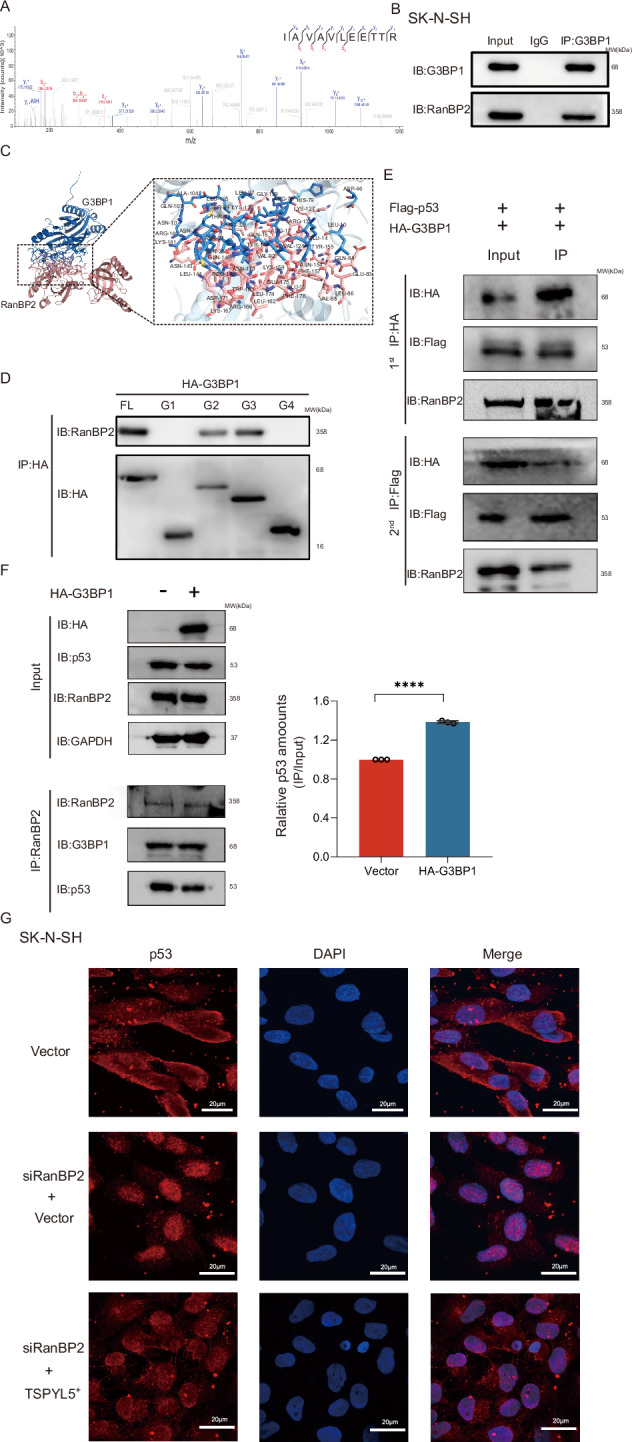
RanBP2 is necessary for the TSPYL5/G3BP1-mediated p53 sumoylation. **A** Mass spectrometry analysis identified RanBP2 in the binding protein pool of G3BP1. **B** Endogenous interaction between G3BP1 and RanBP2 was determined using Co-IP with anti-G3BP1 antibodies in SK-N-SH cells. **C** The binding sites of G3BP1 and RanBP2 proposed by computational docking model. G3BP1 molecule was shown as blue sticks, and RanBP2 was shown as pink sticks. **D** Interaction between the PXXP domain of G3BP1 and RanBP2 was determined using Co-IP with anti-HA antibodies. The G1 to G4 domains of G3BP1 are same to that in Fig. 6D. **E** Two-step Co-IP assays showed the present of RanBP2-G3BP1-p53 complex in SK-N-SH cells. **F** G3BP1 overexpression increased the binding of p53 to RanBP2 in SK-N-SH cells using IP with anti-RanBP2 antibodies. Data are presented as the mean ± SD from 3 experiments, ***p < 0.001. **G** Immunofluorescence analysis showed the nuclear import of p53 in SK-N-SH cells with RanBP2 knockdown. Scale bar = 20 μm.

Sumoylation, like ubiquitination, is also a sequential multienzymatic process in which SUMO E3 ligases play a critical role in the recruitment of substrates and the transfer of SUMOs onto targets [35]. To date, G3BP1 has not been demonstrated to function as an E3 ligase. Second, our work found that the binding of TSPYL5 to G3BP1 seriously disturbed the interaction between p53 and G3BP1 (Supplementary Fig. 10a, b), providing an explanation for the absence of the TSPYL5-G3BP1-p53 complex and suggesting that the binding of TSPYL5 to G3BP1 could not establish a functional platform for p53 sumoylation. In this case, we further explored the E3 sumo-ligases in G3BP1-interacting proteins by Co-IP and LC‒MS/MS assays. The results indicated that RanBP2 was a high-scoring partner of G3BP1 (Fig. 5A). Previous studies have identified that RanBP2 is one of the E3 sumo-ligases for p53 sumoylation [36]. Therefore, we further performed IP and molecular docking assays and confirmed the binding between G3BP1 and RanBP2 in NB cells (Fig. 5B, C). More detailed work indicated that the PXXP domain (from 222 aa to 338 aa) of G3BP1 was necessary for its binding to RanBP2 (Fig. 5D).

Importantly, in the present study, we identified the presence of the RanBP2-G3BP1-p53 complex and the interaction among the three proteins in the complex (Fig. 5E), displaying a functional system potentially performing the sumoylation of p53. Furthermore, we observed that G3BP1 overexpression increased the binding between p53 and RanBP2 (Fig. 5F). Therefore, it is likely that in the RanBP2-G3BP1-p53 complex, G3BP1 acts as a recruiter of p53 to enhance the interaction between RanBP2 and p53 and promote RanBP2-mediated p53 sumoylation. Furthermore, downregulation of the E3 sumo-ligase RanBP2 promoted the nuclear translocation of p53 in SK-N-SH cells with different TSPYL5 levels (Fig. 5G), further supporting that RanBP2 is involved in TSPYL5-improved p53 sumoylation and nuclear export. Here, we are almost certain that G3BP1 and RanBP2 are two crucial members of the underlying mechanism of TSPYL5-regulated p53 sumoylation.

### TSPYL5 enhances the interaction of G3BP1 and RanBP2 by driving the nuclear membrane translocation of G3BP1

**Fig. 6:**
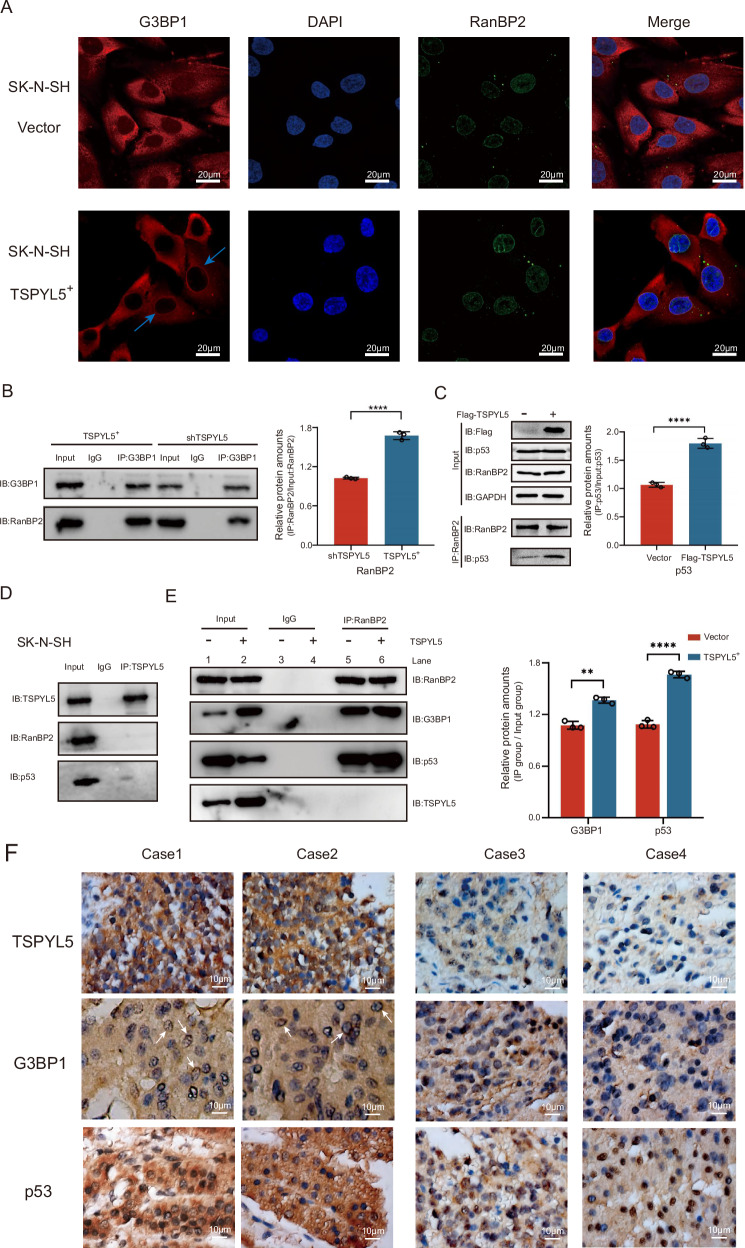
TSPYL5 enhances the nuclear membrane aggregation of G3BP1 to facilitate the formation of RanBP2-G3BP1-p53 complex. **A** Immunofluorescence analysis showed the increase of G3BP1 on the nuclear membrane in SK-N-SH with TSPYL5 overexpression (blue arrows pointed). Scale bar = 20 μm. **B** TSPYL5 overexpression increased the binding of G3BP1 to RanBP2. Nuclear proteins are isolated for Co-IP. Data are presented as the mean ± SD from three experiments, ***p < 0.001. **C** TSPYL5 overexpression increased the binding of RanBP2 to p53. Data are presented as the mean ± SD from three experiments, ***p < 0.001. **D** Co-IP assays showed the absence of TSPYL5-RanBP2 or TSPYL5-p53 complexes in SK-N-SH cells. Nuclear proteins are isolated for Co-IP. **E** Co-IP assays with nucleoproteins showed the increase of the binding of RanBP2 and G3BP1 or p53 in SK-N-SH with TSPYL5 overexpression. **F** Immunohistochemistry assays showed nuclear membrane aggregation of G3BP1 and cytoplasmic p53 sequestration in two cases with abundant TSPYL5 expression (case 1 and case 2), and such a phenotype of G3BP1 and p53 was absent in other two cases showing few TSPYL5 expression (case 3 and case 4). White arrows indicated the nuclear membrane location of G3BP1. Scale bar = 10 μm.

RanBP2 is a key component of the nuclear pore complex [37], but we did not observe a predominant distribution of either TSPYL5 or G3BP1 on the nuclear membrane of NB cells. This raises the question of how cytoplasmic TSPYL5 promotes the interaction of RanBP2 and G3BP1 on the nuclear membrane. Encouragingly, we found that TSPYL5 overexpression promoted the translocation of G3BP1 to the nuclear membrane with colocalization with RanBP2 in SK-N-SH cells (Fig. 6A). Moreover, we observed that TSPYL5 overexpression enhanced the binding between G3BP1 and RanBP2 as well as the binding between RanBP2 and p53 (Fig. 6B, C). These findings reveal that TSPYL5 promotes the formation of the G3BP1/RanBP2/p53 complex by mediating the nuclear membrane accumulation of G3BP1. Nevertheless, we did not observe an interaction between TSPYL5 and RanBP2 or the presence of TSPYL5 in the RanBP2-G3BP1-p53 complex (Fig. 6D), although the binding of TSPYL5 to G3BP1 increased the formation of the RanBP2-G3BP1-p53 complex (Fig. 6E), and the PXXP domain of G3BP1 is necessary for binding to TSPYL5 or RanBP2. Thus, we speculate that TSPYL5 could be displaced by RanBP2 when G3BP1 interacts with RanBP2.

Furthermore, using human neuroblastoma tissues, we analyzed the correlation of the subcellular localization of p53 and G3BP1 with the expression level of TSPYL5. As a result, we observed clearly nuclear membrane aggregation of G3BP1 and cytoplasmic p53 sequestration in five cases of NB with abundant TSPYL5 expression, while such a phenotype of G3BP1 and p53 was absent in other three cases showing few TSPYL5 expression (Fig. 6F, Supplementary Fig. 11). These observations in vivo confirm a decisive role of high TSPYL5 expression in the nuclear membrane aggregation of G3BP1 and cytoplasmic p53 sequestration in NB tumors.

### TSPYL5 promotes casein kinase 2-dependent G3BP1 Ser149 phosphorylation to drive G3BP1 translocation

**Fig. 7:**
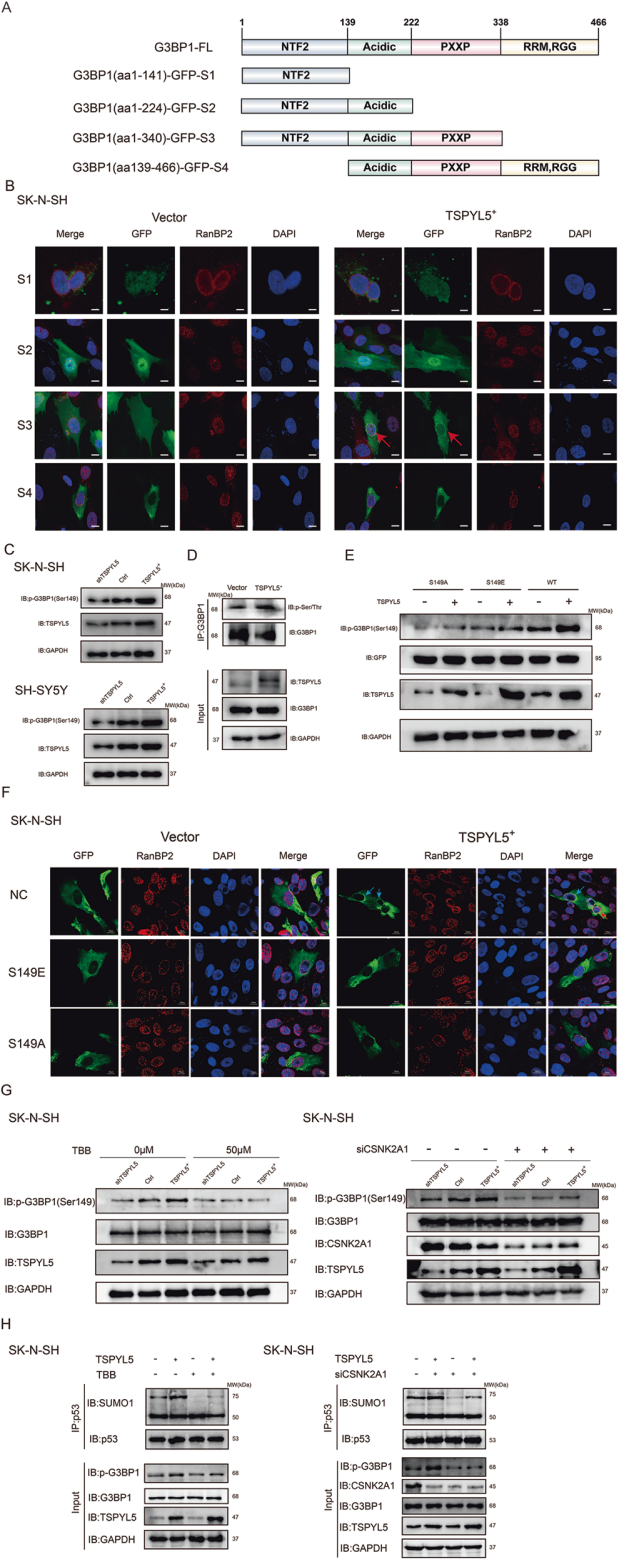
TSPYL5 promotes the phosphorylation of G3BP1 Ser149 to facilitate its nuclear membrane translocation. **A** Schematic diagram of G3BP1 domain and GFP-fused proteins. **B** Immunofluorescence analysis showed the subcellular location of each fused protein in SK-N-SH cells with TSPYL5 overexpression. Red arrows indicated the nuclear membrane location of the fused proteins. Scale bar = 20 μm. **C** Western blotting with specific antibody of anti-phosphorylated Ser149 of G3BP1 showed the increase in the level of G3BP1 Ser149 phosphorylation in SK-N-SH cells with TSPYL5 overexpression. **D** Co-IP assay showed the increase in the total level of G3BP1 phosphorylation in SK-N-SH cells with TSPYL5 overexpression. **E** The promotion of G3BP1 phosphorylation was absent in TSPYL5-overexpressed SK-N-SH cells with S149A or S149E mutant. **F** The promotion of the nuclear membrane aggregation of G3BP1 was absent in TSPYL5-overexpressed SK-N-SH cells with S149A or S149E mutant. Scale bar = 10 μm. **G** The inhibition of CK2 activity via TBB or CSNK2A1 siRNA decreased the phosphorylation levels of G3BP1 Ser149, even TSPYL5 overexpression. **H** The sumoylated p53 levels declined when inhibiting CK2 activity via TBB or CSNK2A1 siRNA, even TSPYL5 overexpression.

To further investigate the mechanism of TSPYL5-driven nuclear membrane aggregation of G3BP1, we constructed fusion plasmids of each structural domain of G3BP1 fused with GFP (green fluorescent protein) (Fig. 7A). Firstly, we examined the subcellular location of G3BP1-GFP in the TSPYL5-knockdown cells. The results showed that the G3BP1-GFP proteins were dispersedly present in the cytoplasm of the TSPYL5-knockdown cells, with some locating around the nuclear periphery (Supplementary Fig. 12). Then, the immunofluorescence signal of G3BP1-GFP appeared to accumulate clearly in the nuclear periphery when TSPYL5 was overexpressed (Supplementary Fig. 12). These results indicated that TSPYL5 enhanced the aggregation of exogenous G3BP1 on the nuclear membrane.

Furthermore, the immunofluorescence analysis in SK-N-SH cells showed that regardless of whether TSPYL5 was overexpressed, PXXP domain-absent polypeptides containing the NTF2L (nuclear transport factor 2-like) domain were mainly localized in the nucleus; the polypeptides containing the NTF2L and PXXP domains showed nuclear membrane aggregation when overexpressing TSPYL5, while the polypeptides without the NTF2L domain were steadily present in the cytoplasm (Fig. 7B). Taken together, these findings suggest that both the NTF2L and PXXP domains of G3BP1 are indispensable for its nuclear membrane aggregation driven by TSPYL5.

The phosphorylation of G3BP1 at Ser149 in the NTF2L domain has been demonstrated to enhance the nuclear translocation of G3BP1 [38]. Undoubtedly, the nuclear import of G3BP1 could increase the chances of its RanBP2-binding retention on the nuclear membrane. Thus, we investigated the influence of the interaction of TSPYL5 with G3BP1 on G3BP1 phosphorylation. The results verified that TSPYL5 positively regulated G3BP1 Ser149 phosphorylation in NB cells (Fig. 7C), which was supported by the observation that the overexpression of TSPYL5 promoted G3BP1 phosphorylation (Fig. 7D). Moreover, we constructed vectors expressing GFP-fused G3BP1 mutants harboring Ser149Ala or Ser149Glu and observed that the mutations disturbed G3BP1 phosphorylation by TSPYL5 (Fig. 7E). Importantly, both mutants were mainly present in the cytoplasm regardless of whether TSPYL5 was overexpressed (Fig. 7F). Moreover, we treated cells with siRNA and chemical inhibitor TBB of casein kinase 2 (CK2) which has been demonstrated to phosphorylate G3BP1 at the site of Ser149 [39]. We found that the inhibition of CK2 activity decreased the levels of TSPYL5-mediated G3BP1 Ser149 phosphorylation (Fig. 7G), and p53 sumoylation followed (Fig. 7H). All of these results support that the interaction of TSPYL5 and G3BP1 promotes CK2-dependent G3BP1 Ser149 phosphorylation which further enhances the nuclear membrane translocation of G3BP1 to form the G3BP1/RanBP2/p53 complex for p53 sumoylation and nuclear export.

## Discussion

**Fig. 8:**
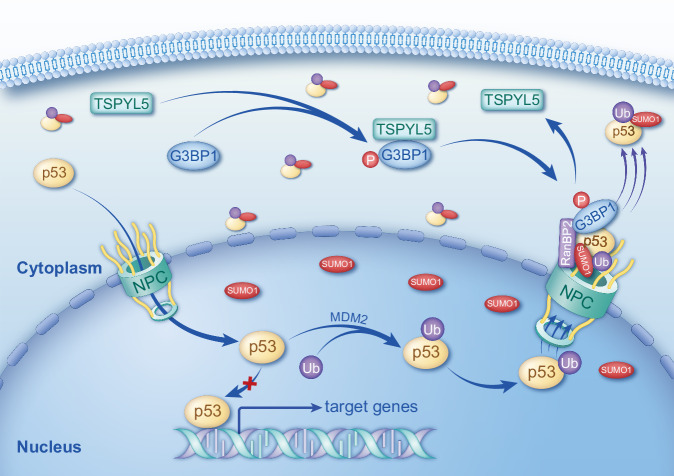
Proposed model of TSPYL5-driven nuclear membrane aggregation of G3BP1 and RanBP2/SUMO1-mediated p53 cytoplasm sequestration in neuroblastoma cells.

Our present study demonstrates a TSPYL5-driven molecular axis positively regulating p53 sumoylation and nuclear export, creating cytoplasmic p53 sequestration in NB cells (Fig. 8). In detail, TSPYL5 binding to the PXXP domain of G3BP1 enhances CK2-dependent G3BP1 Ser149 phosphorylation in the cytoplasm, promoting the NTF2L domain-dependent nuclear import of G3BP1; nucleoporin RanBP2 physically interacts with nuclear-transferred G3BP1, causing the aggregation of G3BP1 on the nuclear membrane; the binding of RanBP2 to G3BP1 dissociates TSPYL5 from G3BP1, recovering the ability of G3BP1 to bind to p53; G3BP1 recruits p53 for RanBP2 at the nuclear pore, facilitating the formation of the RanBP2-G3BP1-p53 complex; the increase in the RanBP2-G3BP1-p53 complex enhances RanBP2-mediated sumoylation of monoubiquitinated p53 in NB cells with low MDM2 levels, accelerating p53 nuclear export; the decrease in nuclear p53 suppresses its transcriptional activity and further lowers MDM2 levels, positively feeding back for p53 monoubiquitination/sumoylation-mediated nuclear export and cytoplasm localization. Our findings uncovered a dynamic model regulating p53 sumoylation, proposing the mechanism underlying p53 cytoplasm sequestration in NB. The suppression of p53 nuclear function by TSPYL5/G3BP1 could provide additional clues for the etiology and treatment of NB cancers, considering the remarkable anti-cancer effect of wild-type p53. Similarly, we suggest that it is of significance to explore whether the TSPYL5-driven mechanism works in other cancers with preponderant cytoplasm localization of p53 and abundant ectopic expression of TSPYL5, including part of breast, liver, and colorectal cancers [40].

Similar to ubiquitination, sumoylation requires E3 ligases to recruit substrates and accelerate the transfer of SUMOs onto targets. Among the dozens of E3 sumo-ligases identified [41], RanBP2 is a noncanonical member due to the absence of a HECT domain or RING finger, two motifs present in most E3 ligases [42]. For this, it seems to be essential that additional partners assist RanBP2 in performing the function of sumoylation. However, little is known about such molecules. Moreover, RanBP2 is a pivotal docking factor of the nucleocytoplasmic transport machinery and is mechanistically involved in the translocation of proteins by mediating sumoylation [43], which also highlights the importance of understanding the system of RanBP2-mediated sumoylation. Undoubtedly, the identification of G3BP1, a well-known nucleating protein of stress granules, as a functional partner of RanBP2 enriches our knowledge of the mechanism of RanBP2-mediated protein sumoylation and translocation. Recent studies demonstrated that G3BP2, a close structural homolog of G3BP1, was also involved in RanBP2-mediated p53 sumoylation in prostate cancer [43, 44]. However, what factors regulate the involvement of G3BP2 in RanBP2-mediated p53 sumoylation and how remain unknown. Our findings provide a logical explanation for the translocation of cytoplasmic G3BP1 to the nuclear pore. Nevertheless, it must be mentioned that G3BP1, rather than G3BP2, interacts with TSPYL5 via PXXP, a domain shared by G3BPs. This may be explained by the large discrepancy in the amino acid sequence between the PXXPs of the two homologs. Therefore, it is not excluded that an unnoticed molecular plays a similar role to TSPYL5 in the nuclear membrane translocation of G3BP2 in prostate cancer cells.

*MYCN* amplification is strongly correlated with advanced stage and failed treatment of NB, so it is recognized as a major grading factor for high-risk NB [45]. For the same reason, various MYCN-targeted strategies are the subject of multiple preclinical trials for high-risk NB [46]. However, the heterogeneity of NB tumors limits the current development of targeted therapy. Actually, *MYCN* amplification is identified in only ~20% of primary NB tumors [47]. Therefore, it is popular to explore more factors applicable to the risk assessment and targeted strategies of NB tumors. The tumor suppressor p53 is often considered a desirable target for cancer therapy [40]. p53 activity was initially claimed to be lacking in many NB tumors due to its cytoplasmic localization, while subsequent studies reported that p53 signal transduction is functional in NB cells [48, 49] and that MDM2 antagonists can activate p53-induced apoptotic signaling in NB by inhibiting the p53-MDM2 interaction [50, 51]. Interestingly, recent studies in ovarian carcinomas have demonstrated that disaggregation of cytoplasmic p53 redistributes it to the nucleus and rescues p53 tumor suppression [52, 53]. These findings imply that the recovery of p53 nuclear localization may restart its suppression of NB. Our present study reveals that TSPYL5 drives p53 cytoplasm localization and that TSPYL5 depletion enhances p53 nuclear translocation and rescues p53 transcriptional activity, suggesting the potential of TSPYL5-targeted strategy in the treatment of NB.

Here, we must mention an unexpected observation, that is, in the present study, the significant impact of TSPYL5 on the prognosis was found only in patients with low or medium-risk NB (Supplementary Fig. 13). Indeed, it is difficult to understand logically the observation due to that the TSPYL5-driven p53 cytoplasm sequestration and transcriptional inactivity also present in the NB cells of patients with high-risk feature. In our opinion, it may result from the difference in the treatment intensity after tumor resection between patients with low/medium and high-risk NB. Due to that X-rays or cytotoxic drugs could increase p53 nuclear translocation [48, 49], the high-intensity chemotherapy and/or radiotherapy after tumor resection may override TSPYL5-driven cytoplasmic p53 retention, impairing the negative influence of TSPYL5 on p53 nuclear activity in patients with high-risk NB. In contrast, for patients with low or medium-risk NB, the low-intensity treatments after tumor resection may not effectively break TSPYL5-mediated p53 suppression, causing an observable effect of TSPYL5 on the prognosis of the patients. If it is the case, it is reasonable to adopt TSPYL5 as a novel risk factor for NB classification to intensify postoperative treatment and improve the prognosis of patients with high TSPYL5 expression. Additionally, we must acknowledge that the limitations that exist in the present study. Firstly, we could not obtain the fresh neuroblastoma tissues in which to investigate the nuclear periphery location of G3BP1 and its interaction with TSPYL5. Secondly, we failed to obtain the tumor-bearing mice using the subcutaneous injection of SK-N-SH or SH-SY5Y cells in BALB/c nude mice, as a result, we did not examine the potential role of TSPYL5 in an in vivo mouse model of NB. Despite all this, the recent patient-derived organoid/xenograft models, acting as advanced tools, will be useful to further explore the biological functions and clinical significance of TSPYL5 in NB.

In conclusion, our study reveals that TSPYL5 uniquely suppresses p53 signaling by promoting G3BP1/RanBP2-mediated p53 sumoylation to tether p53 in the cytoplasm of NB cells. We demonstrate that the abundant ectopic expression of TSPYL5 is not conducive to NB treatment and prognosis, suggesting that the depletion of TSPYL5 and disruption of the interaction between TSPYL5 and G3BP1 may provide interesting therapeutic opportunities for NB patients.

## Materials and methods

### Cell culture

All cell lines used in this study were purchased from the Type Culture Collection of the Chinese Academy of Sciences (Shanghai, China) and maintained in our laboratory. SK-N-SH and SH-SY5Y cells were cultured in Minimum Essential Medium (HyClone, Logan, UT) containing 1% penicillin/streptomycin (HyClone), 10% fetal bovine serum (FBS) (Gibco, Carlsbad, CA), 1% nonessential amino acids (Procell, Wuhan, China), and 1% sodium pyruvate (Procell). Other cells were grown in RPMI-1640 (HyClone) medium supplemented with 10% FBS and 1% penicillin/streptomycin. Cells were incubated at 37 °C in a humid atmosphere with 5% CO_2_ and 95% air.

### Chemicals and antibodies

All chemicals for the experiments were reagent grade or better. The protease inhibitor, MG132, was provided by Selleck (Billerica, MA). Cisplatin and 4,5,6,7-tetrabromobenzotriazole (TBB) were obtained from MedChemExpress (MCE, Shanghai, China). Verapamil and Hoechst 33342 were purchased from Solarbio (Beijing, China). Arsenate was provided by Sigma-Aldrich (St. Louis, MO). Details of the specific antibodies used in this study are listed in Supplementary Table 1.

### DNA constructs and mutagenesis

The cDNA of *TSPYL5*, *G3BP1, TP53*, and *SUMO1* were separately synthesized and cloned and inserted into a pcDNA^TM^3.1^(+)^ vector (Invitrogen, Carlsbad, CA) containing a tag sequence of FLAG, HA or Myc. Three lentiviral vectors for overexpressing, knocking down, and recovering TSPYL5 expression were constructed by GeneCopoeia (Rockville, MD). Additionally, the full-length cDNA of *TSPYL5* was also cloned and inserted into a pLVX-ZsGreen1-N1 vector (GeneCopoeia). *TSPYL5*-specific shRNA (shTSPYL5) was synthesized and inserted into the pLKO.1 vector (GeneCopoeia). All siRNAs used in this study were synthesized by GenePharma Co., Ltd. (Shanghai, China). The sequences of the siRNAs are listed in Supplementary Table 2. Three mutant expression vectors, including p53-K386R, G3BP1-S149A, and G3BP1-S149E, were generated by PANOMIX Biomedical Tech Co., LTD (Suzhou, China).

### Immunoprecipitation (IP) and western blotting

Cells were lysed with lysis buffer containing protease inhibitor cocktail (Bimake, Carlsbad, CA) and phosphatase inhibitor cocktail (Bimake), followed by centrifugation at 4 °C. The supernatants were immunoprecipitated with the indicated antibodies or IgG and incubated overnight at 4 °C. Then, the mixture was additionally incubated with Protein A+G Agarose beads (Beyotime, Shanghai, China) and rotated for 2 h at 4 °C. After washing five times with lysis buffer, the beads were mixed with SDS‒PAGE loading buffer and boiled for 8 min. After separation by SDS–PAGE, the proteins were transferred onto polyvinylidene difluoride membranes (Millipore, Billerica, MA). Following the blocking procedure, membranes were reacted with primary antibodies overnight at 4 °C. The membranes were washed three times and incubated with secondary antibodies for 2 h at room temperature. Specific proteins were visualized using an Immobilon™ chemiluminescence western blotting detection system (Millipore).

### Liquid chromatography-tandem mass spectrometry (LC‒MS/MS)

IP samples were separated by SDS–PAGE, and protein bands were excised from the gel. These protein fragments were washed twice with 100 mM bicarbonate in acetonitrile and then digested with trypsin. Then, 0.1% formic acid was added to the supernatants, which were subjected to LC‒MS/MS analysis with an Orbitrap-Fusion mass spectrometer (Thermo Scientific, Waltham, MA) accompanied by Easy-nLC 1000 (Thermo Scientific). Datasets were generated from at least three independent experiments. The raw data derived from the LC‒MS/MS analysis were used in Proteome Discoverer 2.4 (Thermo Scientific).

### Transcriptomic analysis

Total RNA was isolated using TRIzol reagent (Invitrogen). After determining the quality and concentration using an Agilent 2100 bioanalyzer (Agilent Technologies, Santa Clara, CA), RNAs were reverse transcribed and sequenced in a HiSeq6000 instrument (Illumina, San Diego, CA). Triplicate RNA samples of each group were prepared for sequencing. The bioinformatic analysis was carried out by Genedenovo Biotechnology Company (Guangzhou, China). Gene Ontology (GO) analysis was visualized for annotation, visualization, and integrated discovery by using the database (https://david.ncifcrf.gov/). GeneRatio was calculated by the percentage of differentially expressed genes (DEGs) that matched a specific GO term in the total DEGs. Kyoto Encyclopedia of Genes and Genomes (KEGG) pathway enrichment analysis was performed by using the KEGG Orthology-based Annotation System 2.0 (KOBAS 2.0, http://kobas.cbi.pku.edu.cn).

### Single-cell RNA-seq data reanalysis

The single-cell RNA-seq data of 14 patients with NB were obtained from the GEO (GSE137804) database, and the clinical characteristics of the samples were collected from the report [17]. After removing cells with a gene count of 500–6000, a unique molecular identifier (UMI) below 30,000, or more than 20% mitochondrial content, a total of 164,296 cells were used for subsequent analyses. Seurat v2.3 was used for dimension reduction and clustering. NormalizeData and ScaleData were used to normalize and scale all gene expression values. The top 2,000 variable genes were selected for principal component analysis (PCA) by FindVariableFeatures. Using the top 20 principal components and a resolution parameter of 0.5, the cells were separated into six clusters by the FindClusters function. For two-dimensional visualization, the RunUMAP function generated uniform manifold approximation and projection (UMAP) plots by the same PCs (principal components) and default settings.

### Reverse transcription-quantitative PCR (RT-qPCR)

Total RNA was reverse transcribed into cDNA using a RevertAid First-Strand cDNA Synthesis Kit (Thermo Scientific). Using SYBR Green Master Mix, quantitative PCR was performed in an iCycler IQ™ system (Bio-Rad, Hercules, CA). Each assay was carried out in triplicate. *GAPDH* was used as an internal control. The RT‒qPCR primers for target genes are listed in Supplementary Table 3.

### Cell proliferation, invasion, and migration assays

For cell proliferation assays, cells were seeded into a 96-well plate (3000 cells/well) and incubated overnight. The next day, cells were treated with 10 μM cisplatin. Using the Cell Counting Kit-8 (Vazyme, Nanjing, China), cell numbers were measured every 24 h four times. Cell invasion assays were performed with Matrigel-coated chambers (BD Biosciences, Franklin Lakes, NJ). After staining, cells were counted using a light microscope in four randomly selected fields. Cell migration was examined by using wound healing migration assays. The migration areas were calculated using Prism 8 software.

### Colony formation assay

Cells were seeded into 35-mm plates with a density of 1000 cells per well for colony formation assay. After culturing for 10–14 days, cells were washed by PBS, fixed with formaldehyde, and stained with 0.5% crystal violet (Biosharp, Shanghai, China) for 30 min at room temperature. Colonies with more than 50 cells were counted manually and photographed.

### Sphere formation assay

Cells were cultured in stem cell-permissive medium (Gibco), containing epidermal growth factor (20 ng/mL), basic fibroblast growth factor (20 ng/mL), and B27 serum-free supplement. Suspended cells were seeded into ultralow-attachment 96-well plates (Jet Bio-Filtration, Guangzhou, China) at a density of 1 or 2 cells/well and incubated at 37 °C for 24 h. Then, each well was visually checked for the presence of a single cell. After 10–14 days, spheres were quantitated and photographed under an Olympus IX71 fluorescent microscopy (Olympus, Tokyo, Japan).

### Flow cytometry assay

For cell apoptosis analysis, cells were seeded in 6-well plates in MEM medium with 10% FBS for 24 h and treated with cisplatin. Apoptosis of cells was assessed according to the instructions of the Annexin V-Alexa Fluor 647/PI apoptosis detection kit (BD Pharmacy). Apoptotic cells were identified as both annexin V^+^/PI^−^ and annexin V^+^/PI^+^.

For side population assay, cells were harvested and resuspended at a concentration of 1 × 10^6^ cells/ml and then given 75 µM verapamil (Sigma-Aldrich) for 30 min. The DNA binding dye, Hoechst 33342 (Sigma-Aldrich), was then added to a final concentration of 5 μg/ml and incubated for 90 min in the dark with interval mixing. Cells were then resuspended in ice-cold Hank’s balanced salt solution. PI was added 10 min before sorting to a final concentration of 2 μg/ml.

### Immunofluorescence staining and confocal imaging

For immunofluorescence analysis, cells were cultured on circular coverslips (Biosharp) in 24-well plates, fixed with 4% paraformaldehyde for 15 min, permeabilized with 0.1% Triton X-100 in phosphate-buffered saline (PBS) for 10 min, and incubated with 1% BSA for 1 h at room temperature. Following incubation with primary antibodies at 4 °C overnight, the cells were washed three times with PBS and incubated with Alexa Fluor 488-/555-/647-labeled secondary antibodies (Thermo Scientific). 4′,6-diamidino-2-phenylindole (DAPI, Solarbio) was used for nuclear staining. Images were visualized by an orthostatic two-photon confocal microscope (Nikon, Tokyo, Japan). α-Tubulin, RanBP2, and DAPI (4′,6-diamidino-2-phenylindole) are used separately as the markers of cytoplasm, nuclear membrane, and nucleus in cells.

### Immunohistochemistry

A tissue microarray containing 22 NB specimens was obtained from Bioaitech (Xian, China). The slide was deparaffinized, rehydrated, blocked with 3% H_2_O_2_, and separately incubated with primary antibodies, including anti-TSPYL5, anti-G3BP1, and anti-p53. After washing in PBS, slides were incubated in horseradish peroxidase–conjugated goat anti-rabbit IgG (Proteintech, Wuhan, China) followed by incubation with 3,3′-diaminobenzidine (DAB) substrate (AbsinBioscience, Shanghai, China) and counterstaining with hematoxylin solution. Preimmune rabbit serum was used as a negative control for primary antibodies. The slides were examined under a microscope (AX10 imager, Zeiss, Oberkochen, Germany). Among the 22 NB specimens, only eight were observed the signals of TSPYL5, G3BP1, and p53, simultaneously, while the complete signals of the three proteins could not be obtained in other specimens due to the destruction of their tissues.

### Statistical analysis

GraphPad Prism 8 software was used for statistical analysis. All data from at least three independent experiments are expressed as the mean ± SD. Statistical significance between any two groups was tested using Student’s *t*-test. *P* < 0.05 was considered statistically significant.

## Supplementary information

Supplementary Figures and Tables

Original data for Blots-Figure

Original data for Blots-Supplementary Figure

## Data Availability

RNA sequencing data have been deposited to the Gene Expression Omnibus (GEO) and can be accessed using the following identifiers: GSE223866. Other single-cell RNA sequencing data used in this study are publicly available and can be accessed from GEO for the GSE137804. Additional data related to this paper may be requested from the corresponding author.
