# Supplementary material for: TSPYL5-driven G3BP1 nuclear membrane translocation facilitates p53 cytoplasm sequestration via accelerating RanBP2-mediated p53 sumoylation and nuclear export in neuroblastoma
Source: Cell Death Dis. 2025 May 3;16(1):358. doi: 10.1038/s41419-025-07694-x (PMC12049415; doi:10.1038/s41419-025-07694-x)
Supplement: Supplementary file 1 — Supplementary Figures and Tables [file 41419_2025_7694_MOESM1_ESM.docx]

**Supplementary information**


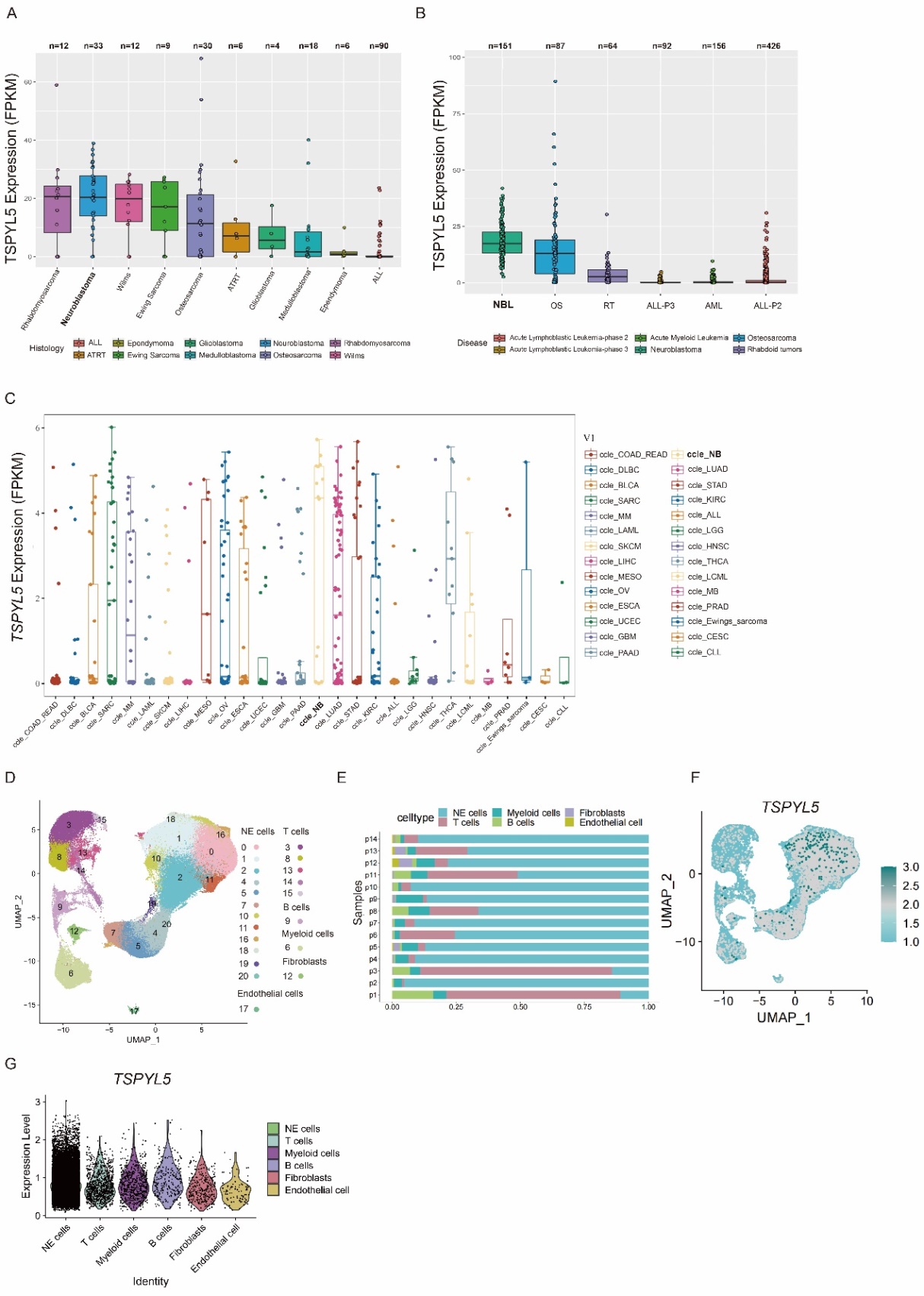
 **Supplementary fig. 1 TSPYL5 is abundantly expressed in NB tumors.**

**A,B** The expression levels of TSPYL5 in ten different pediatric tumors from the PCAT database (A) and six different pediatric tumors from the TARGET database (B). ALL: Acute Lymphoblastic Leukemia. ATRT: atypical teratoid/rhabdoid tumor. **C** The expression levels of TSPYL5 in different cancer cell lines. COAD_READ: Colon adenocarcinoma/Rectum adenocarcinoma Esophageal carcinoma, DLBC: Lymphoid Neoplasm Diffuse Large B-cell Lymphoma, BLCA: Bladder Urothelial Carcinoma, SARC: Sarcoma, MM: multiple myeloma, LAML: Acute Myeloid Leukemia, SKCM: Skin Cutaneous Melanoma, LIHC: Liver hepatocellular carcinoma, MESO: Mesothelioma, OV: Ovarian serous cystadenocarcinoma, ESCA: Esophageal carcinoma, UCEC: Uterine Corpus Endometrial Carcinoma, GBM: Glioblastoma multiforme, PAAD: Pancreatic adenocarcinoma, NB: neuroblastoma, LUAD: Lung adenocarcinoma, STAD: Stomach adenocarcinoma, KIRC: Kidney renal clear cell carcinoma, ALL: Acute Lymphoblastic Leukemia, LGG: Brain Lower Grade Glioma, HNSC: Head and Neck squamous cell carcinoma, THCA: Thyroid carcinoma, LCML: Chronic Myelogenous Leukemia, MB: medulloblastoma, PRAD: Prostate adenocarcinoma, CESC: Cervical squamous cell carcinoma and endocervical adenocarcinoma, CLL: Chronic lymphocytic leukemia. **D** UMAP plot showing major cell types of 14 NB samples classified with single-cell RNA-seq data from GEO (GSE137804). **E** Relative ratio of different type of cells of the 14 NB samples. **F** UMAP plot showing the *TSPYL5* expression pattern in different type of cells of 14 NB samples. **G** Violin plots of *TSPYL5* in different type of cells of 14 NB samples.


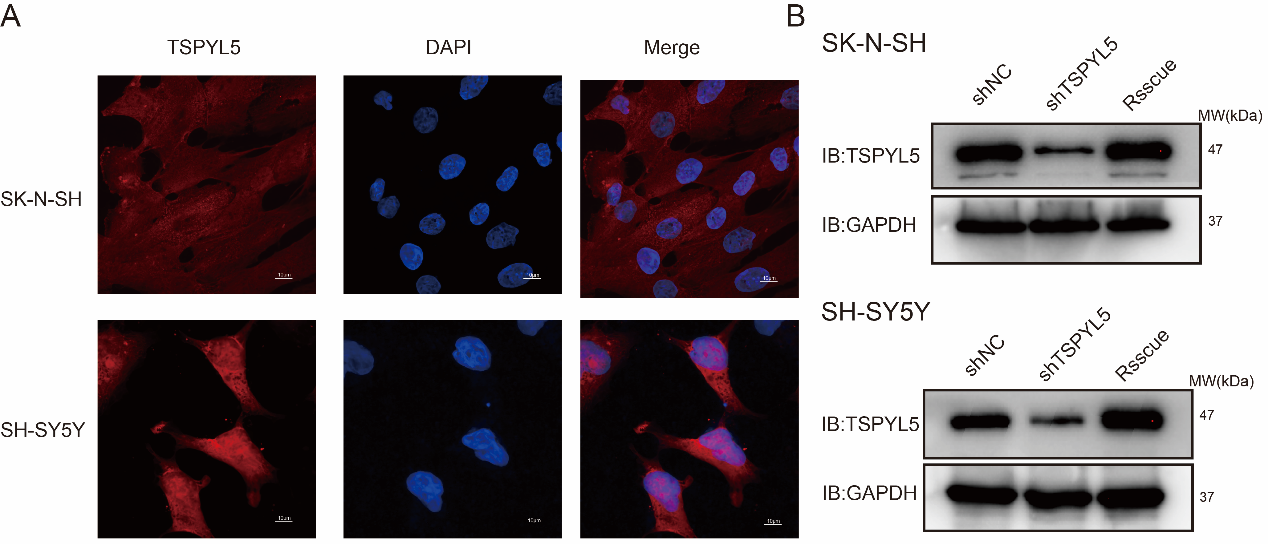


**Supplementary fig. 2 The expression of TSPYL5 in NB cells.**

**A** Immunofluorescent analysis of TSPYL5 location in SK-N-SH and SH-SY5Y cells. **B** Identification of the constructed cell models (TSPYL5 knockdown and rescue) by immunoblotting analysis.


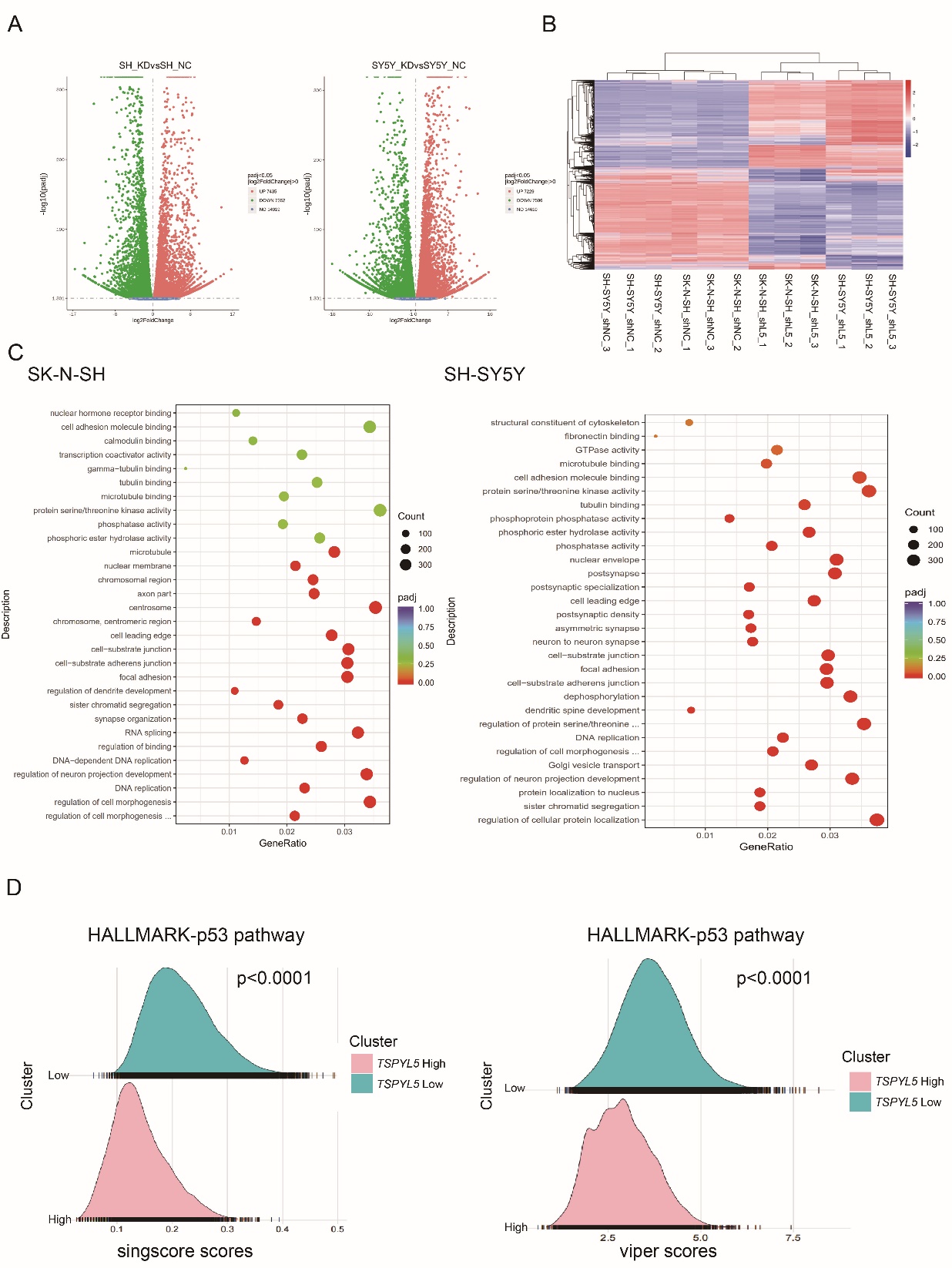


**Supplementary fig. 3 RNA-seq analysis of the SK-N-SH and SH-SY5Y cells with TSPYL5 kncokdown.**

**A** Volcano maps showing the different expression genes (DEGs) derived from RNA-seq. **B** Heatmap of the DEGs showing the similar expression pattern between TSPYL5-kncoked down SK-N-SH and SH-SY5Y cells. **C** GO enrichment analysis of the DEGs showing the potential TSPYL5-related cell functions in SK-N-SH and SH-SY5Y cells. **D** Investigation of the correlation between TSPYL5 expression and p53 signaling function (Hallmark p53 gene set) using single cell RNA-seq data (GSE137804).


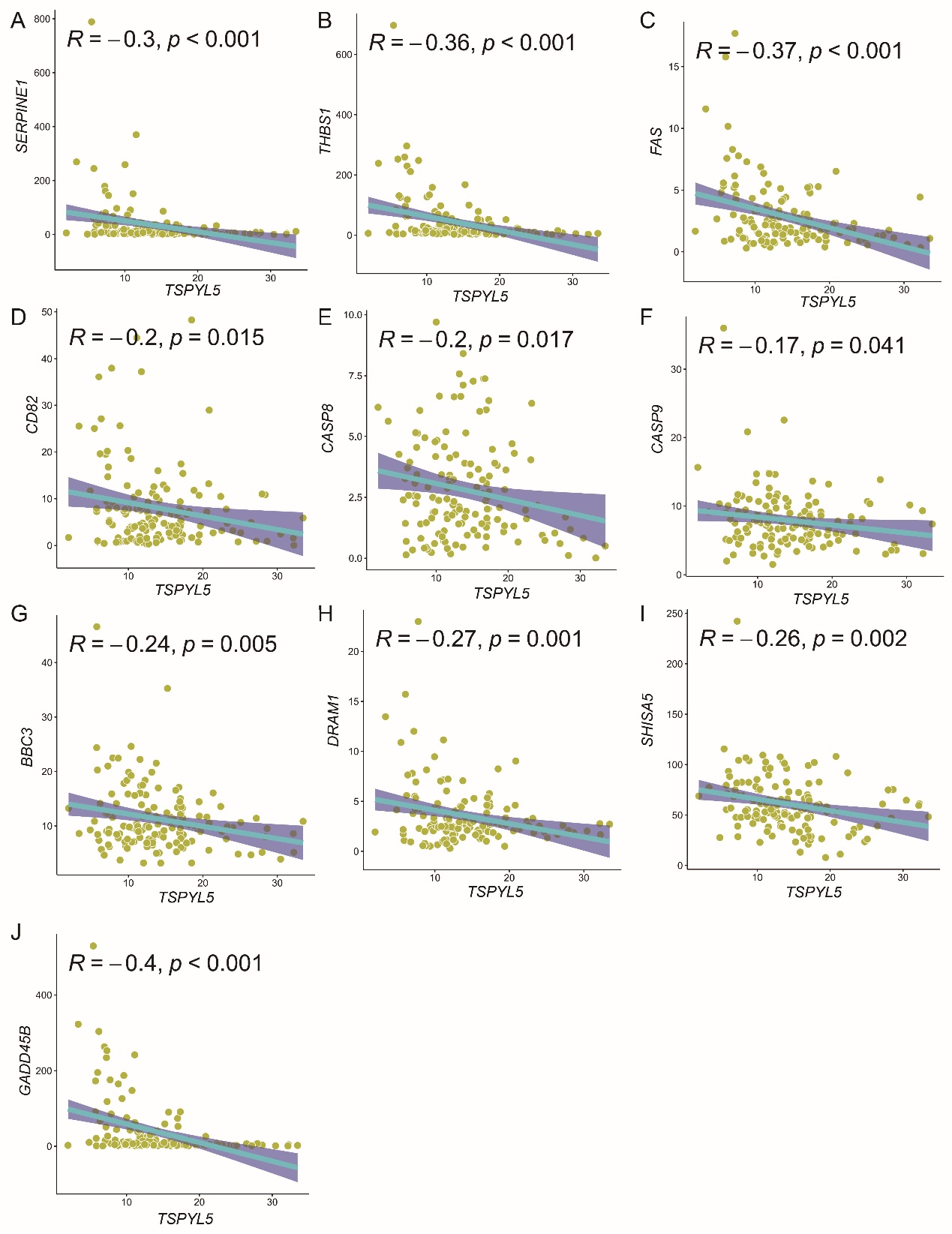


**Supplementary fig. 4 Correlation analysis between the expression of *TSPYL5* and 10 p53-targeted genes (A-J) in the NB tissues.**


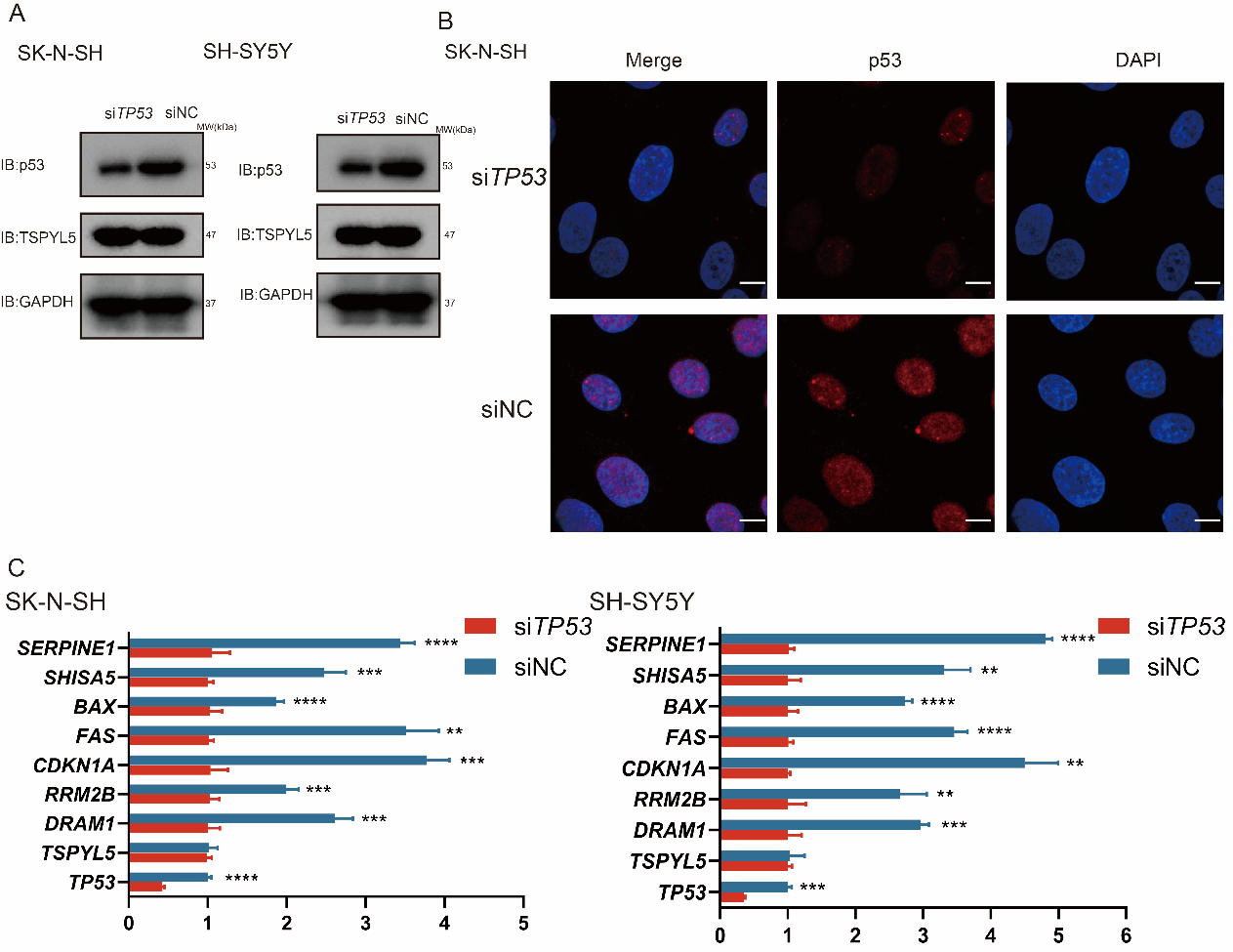


**Supplementary fig. 5 Downregulation of p53 expression in the TSPYL5-knockdown cells**

A, B Identification of p53-knockdown using Western blotting (A) and immunofluorescence assays (B). **C** The p53-knockdown obviously decreased the expression of seven p53-directly targeted genes. The mean ± SD from three experiments was plotted. * p  <  0.05, ** p  <  0.01, *** p  <  0.001.


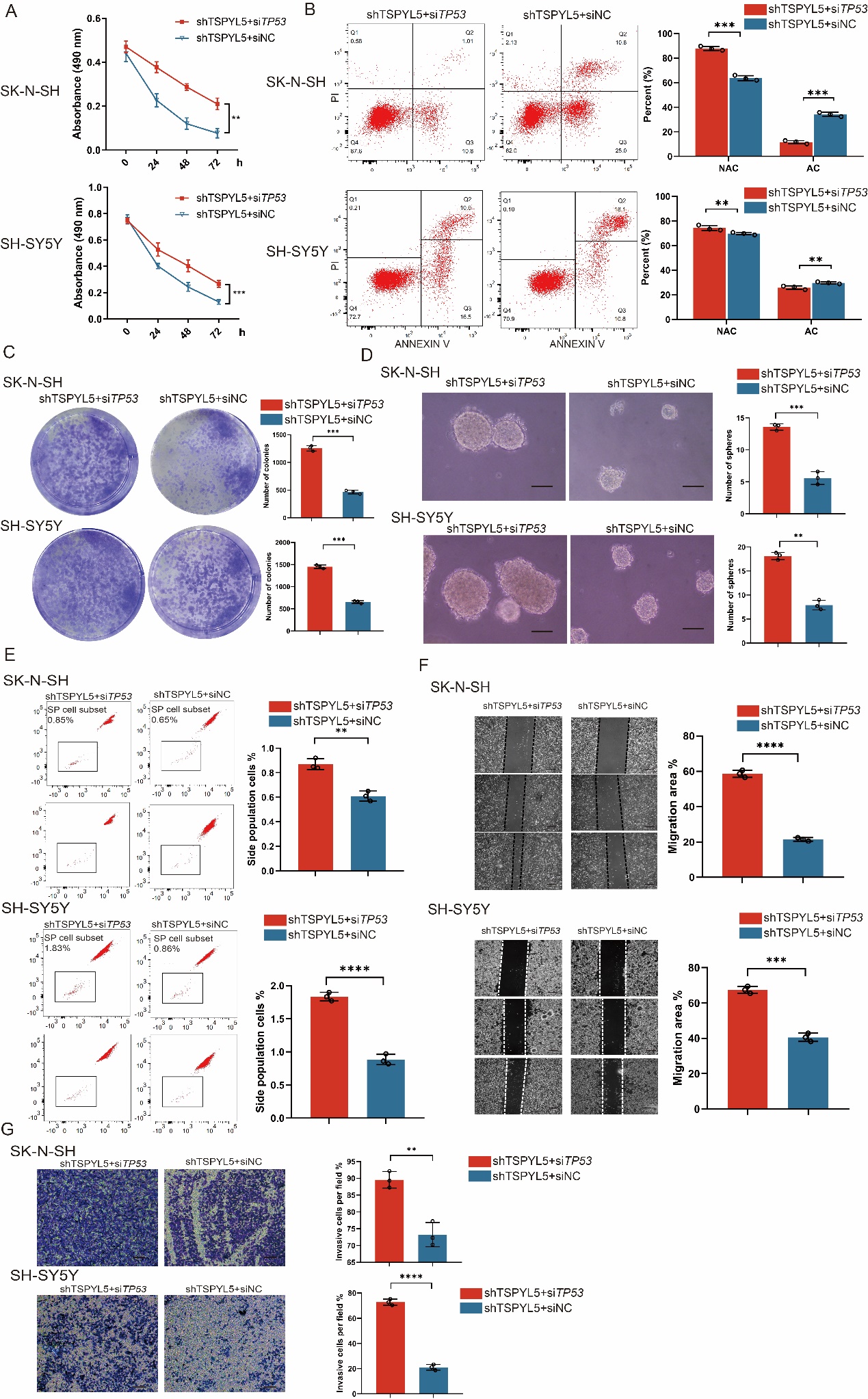


**Supplementary fig. 6 The downregulated p53 expression augments the malignant characteristics of the TSPYL5-knockdown NB cells**

**A** CCK‐8 assays showing the sensitivity to cisplatin in the cells transfected with siTP53 and shTSPYL5. **B** Flow cytometry assays showing the apoptosis induced by cisplatin in the cells transfected with siTP53 and shTSPYL5. **C** Colony formation assays in the cells transfected with siTP53 and shTSPYL5. **D** Sphere formation assays in the cells transfected with siTP53 and shTSPYL5. Scale bar = 25 μm. **E** Side population ratio assays in the cells transfected with siTP53 and shTSPYL5. **F, G** Migration and invasion assays in the cells transfected with siTP53 and shTSPYL5. Scale bar = 50 μm. The mean ± SD from three experiments was plotted. ***p*  <  0.01, ****p* <  0.001, and **** *p* <  0.0001.


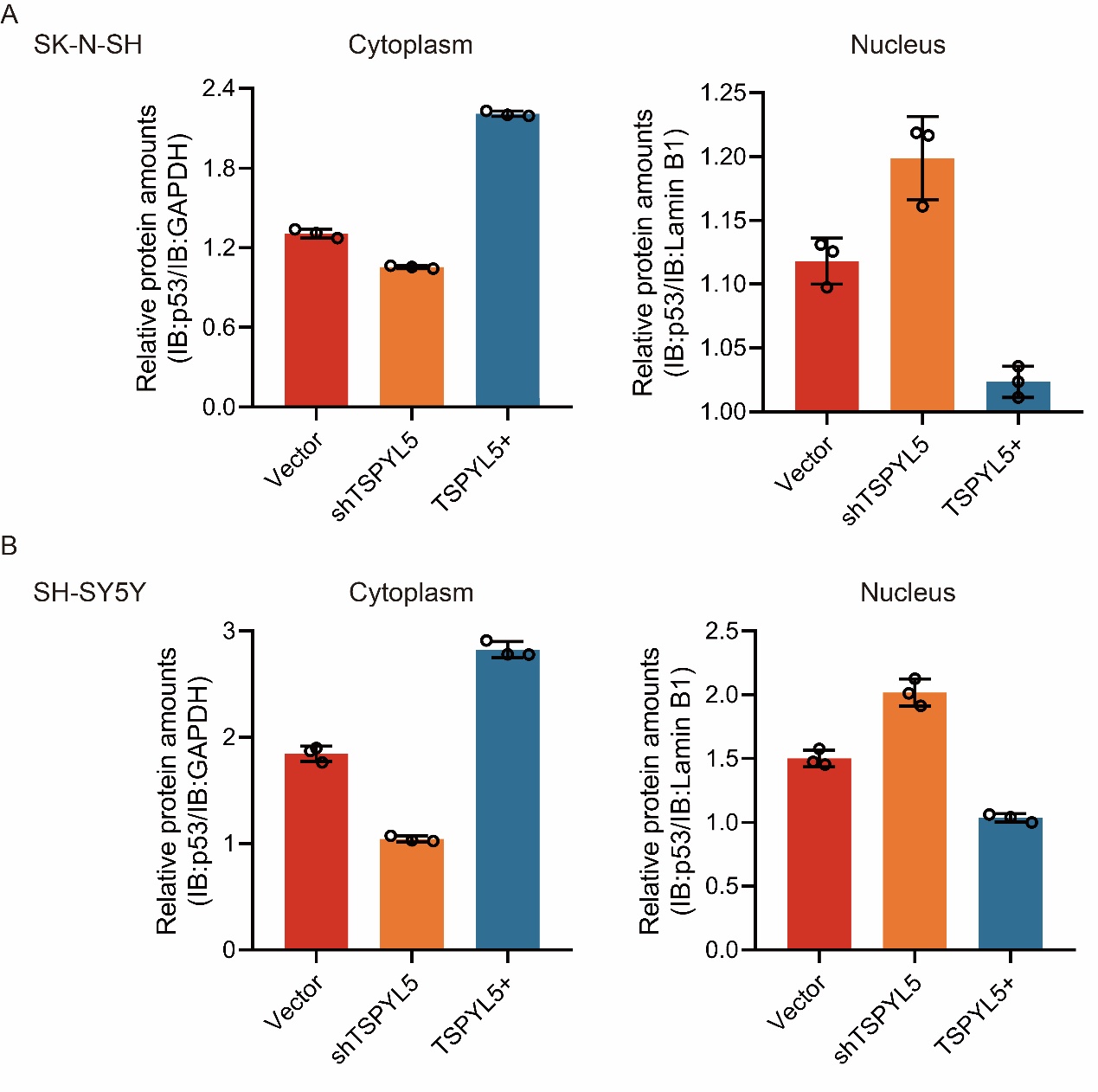


**Supplementary fig. 7 The relative protein level of p53 in nucleus and cytoplasm using the software of Image J to quantify the nucleus-plasm separation assay in Figure 3C.**

**
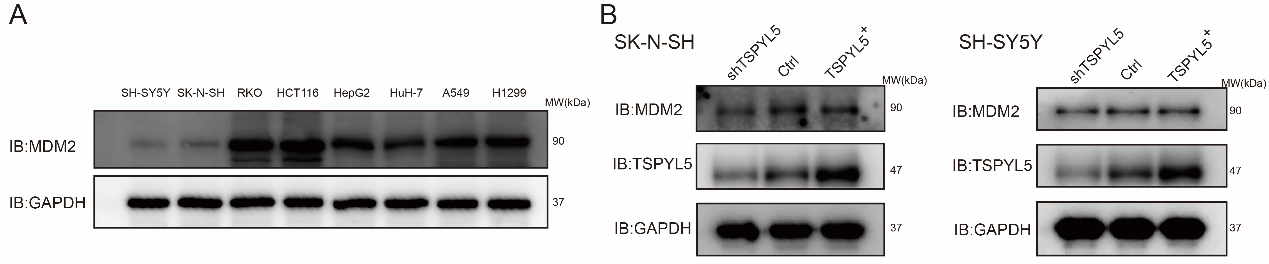
**

**Supplementary fig. 8 Expression levels of MDM2 in different cell lines**

**A** The comparision of MDM2 expression between SK-N-SH/SH-SY5Y and other six cells reported high expression of MDM2. **B** The influence of TSPYL5 on MDM2 expression in SK-N-SH and SH-SY5Y cells


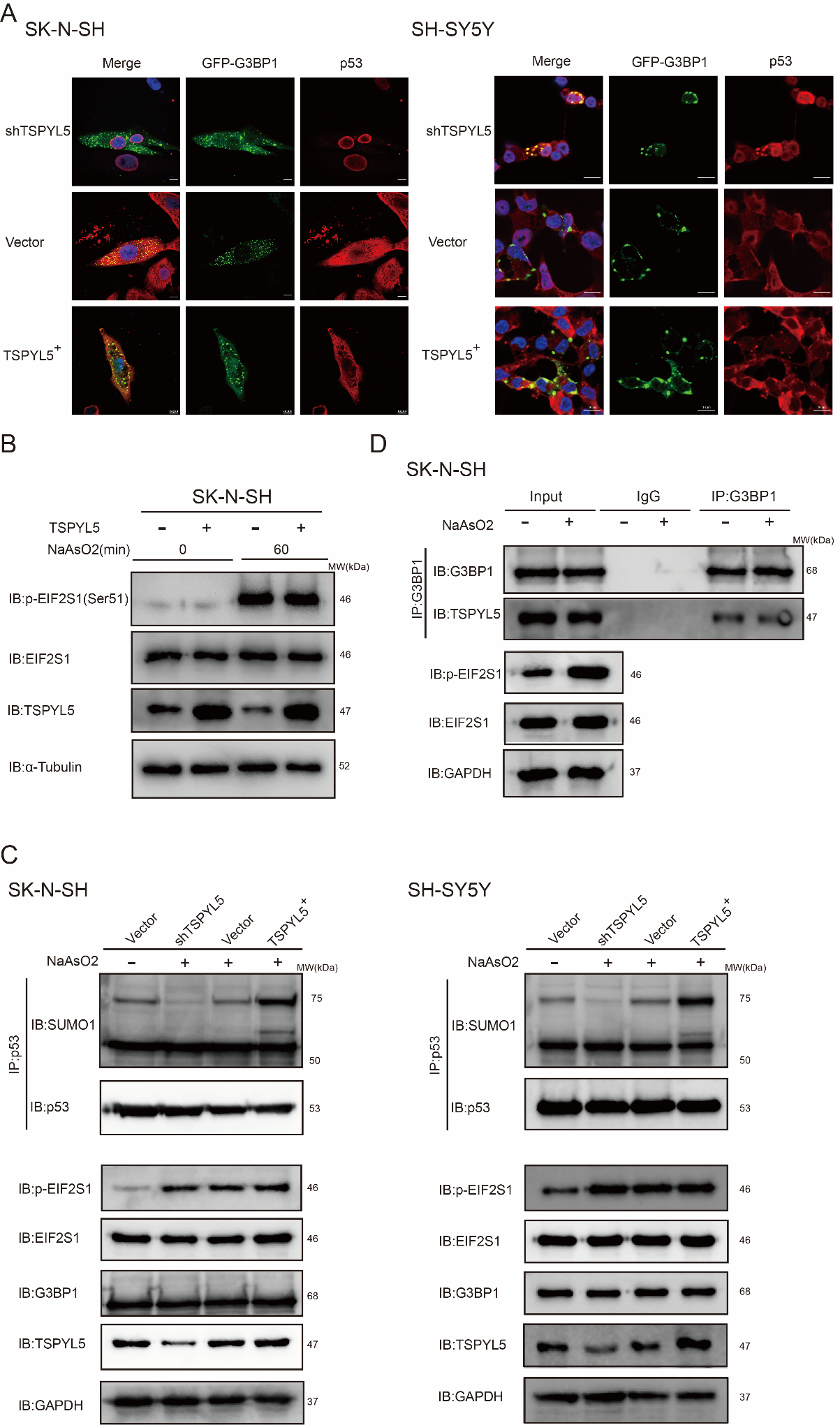


**Supplementary fig. 9 The formation of stress granules (SGs) not interrupts TSPYL5/G3BP1-mediated p53 sumoylation.**

**A** TSPYL5 enhances p53 cytoplasm location in NB cells treated with arsenate. **B** TSPYL5 not influences the phosphorylation level of EIF2S1 in SK-N-SH cells treated with arsenate. **C** SGs formation did not interrupt the TSPYL5-increased p53 sumoylation. **D** The interaction of TSPYL5 with G3BP1 is untouched in SK-N-SH cells treated with arsenate.


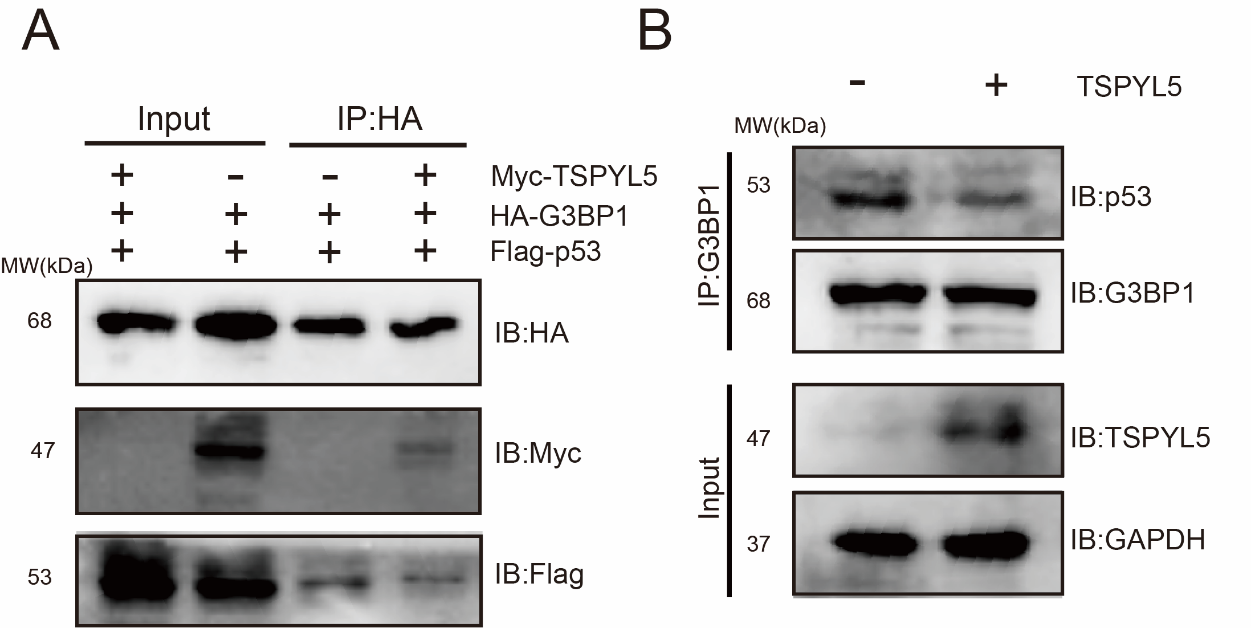


**Supplementary fig. 10 The TSPYL5-G3BP1-p53 complex is absent in NB cells. A** Co-IP assays showed the impairment of the binding of G3BP1 to p53 by TSPYL5 using an *in vitro* transcription and translation system. **B** Co-IP assays showed the impairment of the binding of G3BP1 to p53 by TSPYL5 using a procaryotic expression system.


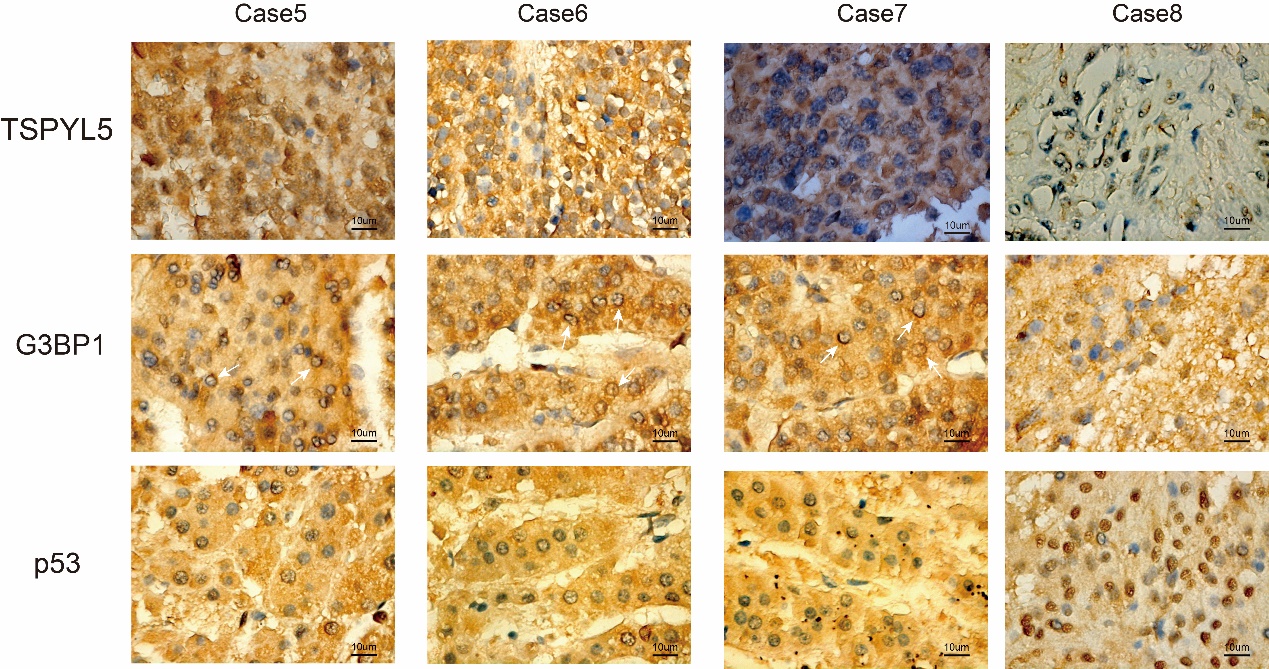


**Supplementary fig. 11 Association of the localization of G3BP1 and p53 with TSPYL5 expression level.** Immunohistochemistry assays showed nuclear membrane aggregation of G3BP1 and cytoplasmic p53 sequestration in three cases of NB with abundant TSPYL5 expression (case 5, case 6 and case 7), and such a phenotype of G3BP1 and p53 was absent in other one case showing few TSPYL5 expression (case 8). White arrows indicated the nuclear membrane location of G3BP1. Scale bar = 10 μm.


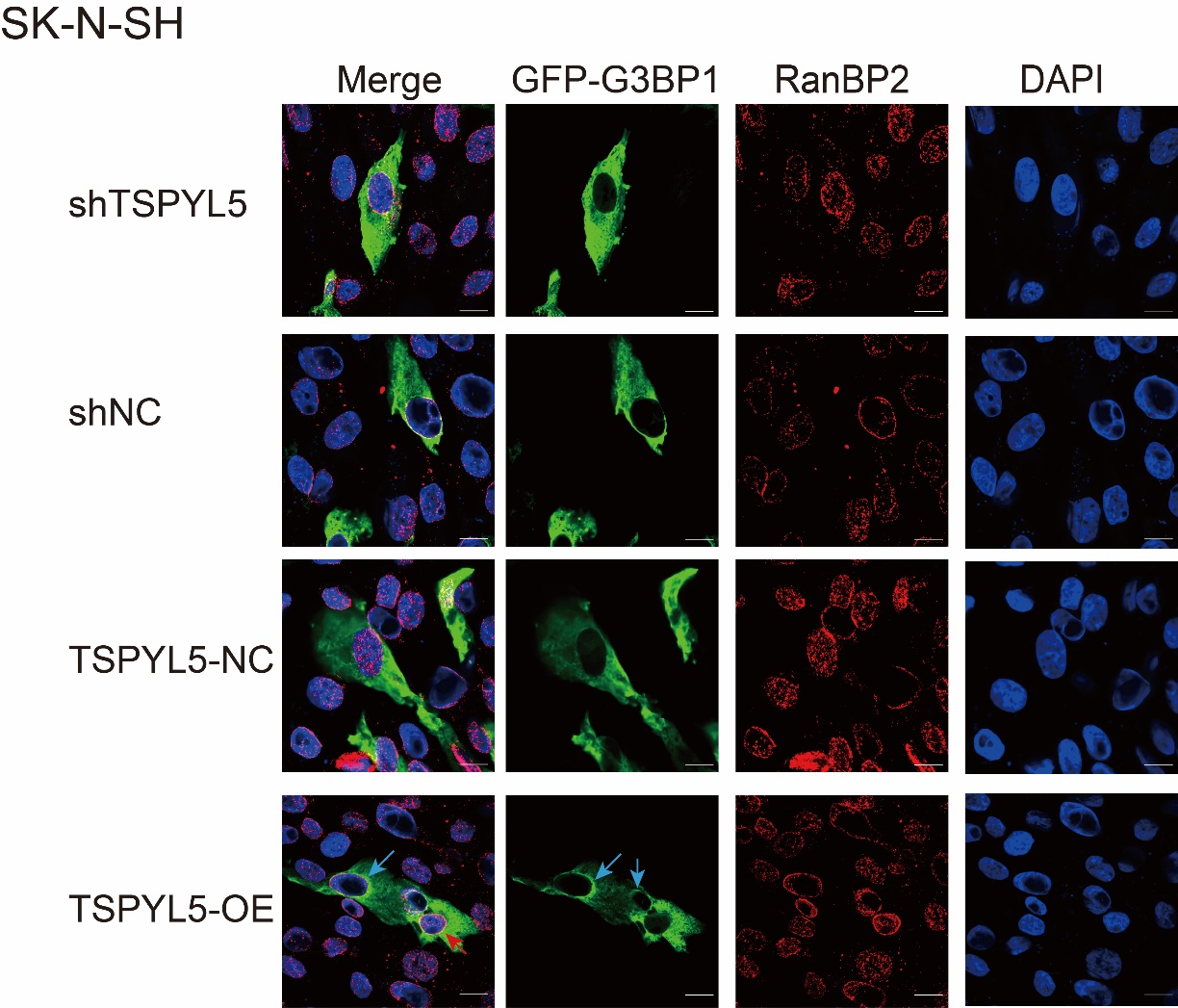


**Supplementary fig. 12 The exogenous G3BP1 expression and location in the cells with TSPYL5-knockdown or –overexpression** (The results of TSPYL5-overexpression and its vehicle control are repeatedly displayed in Figure 7F)


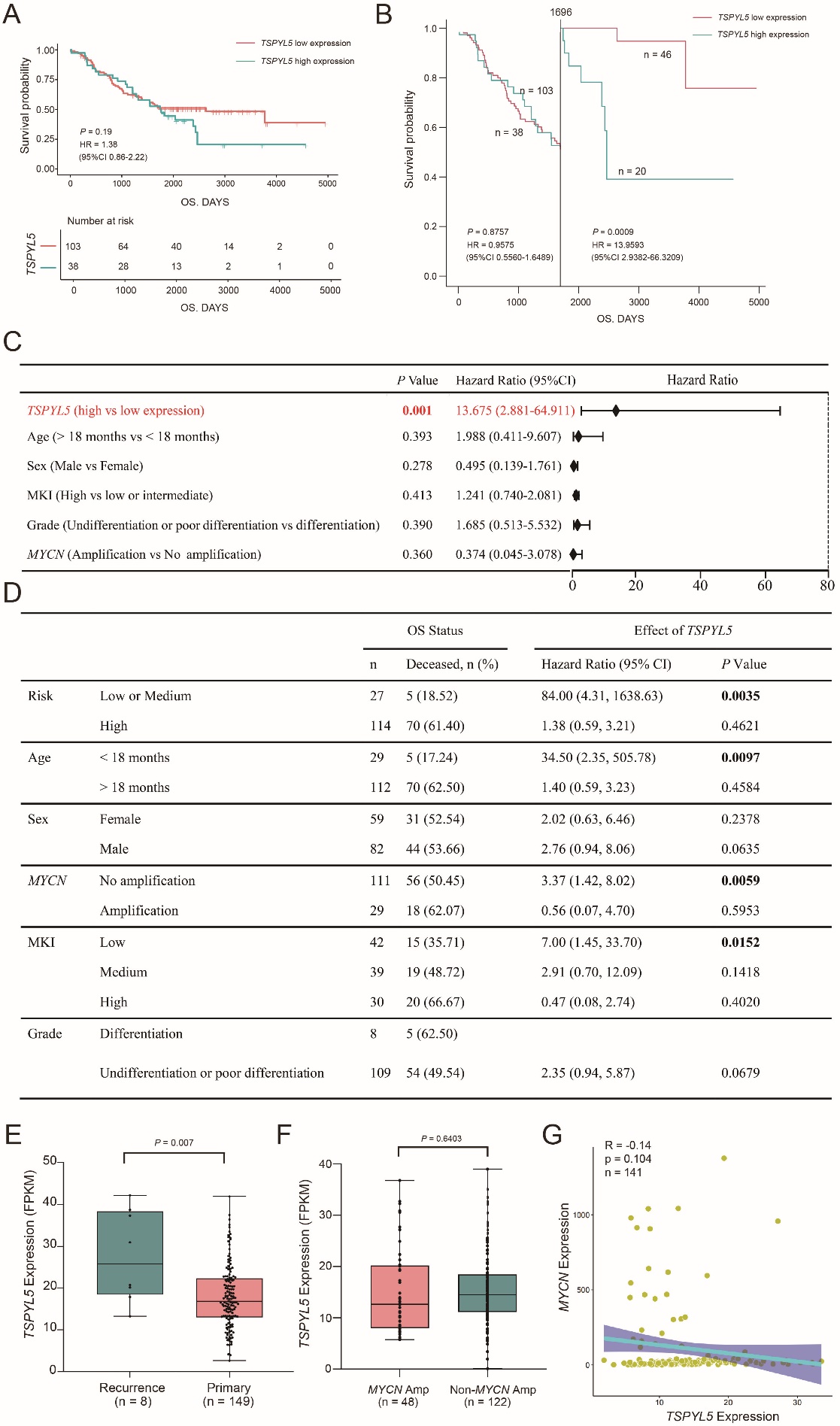


**Supplementary fig. 13 TSPYL5 is correlated with poor prognosis of NB**

**A** Kaplan-Meier curve for the overall survival of NB patients with low and high *TSPYL5* expression. **B** Landmark analysis for the overall survival of NB patients with low and high *TSPYL5* expression. **C** Cox multivariate analysis of *TSPYL5* expression and various clinicopathological indexes including age, sex, MKI, grade and *MYCN* amplification in NB. **D** Stratified analysis of the effect of *TSPYL5* on the overall survival of NB patients with different risk indices. **E** The comparison of *TSPYL5* expression between the patients with recurrent and primary NB. **F** The *TSPYL5* expression level was not significantly different between NB patients with *MYCN* amplification and *MYCN* nonamplification. The data of the NB tumors was obtained from TARGET and PCAT. Amp: Amplification. **G** No significant correlation between the expression levels of *TSPYL5* and *MYCN* in the NB tumors.

Survival analysis was conducted by the Kaplan–Meier method. Landmark analysis was used to determine the endpoint for dividing the short and long-term survival times. Cox multivariate analysis was used to investigate the role of *TSPYL5* in the long-term prognosis of NB patients. Stratified analyses of the clinical characteristics of NB were conducted to test the correlation between the *TSPYL5* expression level and the long-term prognosis of NB patients.

**Supplementary table 1 List of antibodies used in this study**

| **Antibodies** | **Source** | **Identifier** |
| --- | --- | --- |
| TSPYL5 | Santa Cruz Biotechnology | sc-98186 |
| G3BP1 | Proteintech Group, Inc | 13057-2-AP |
| G3BP1 | Santa Cruz Biotechnology | sc-365338 |
| p53 | Proteintech Group | 10442-1-AP |
| p53 | Proteintech Group | 60283-2-Ig |
| Lamin B1 | Proteintech Group | 12987-1-AP |
| GAPDH | Proteintech Group | 10494-1-AP |
| SUMO1 | Proteintech Group | 10329-1-AP |
| SUMO1 | Proteintech Group | 67559-1-Ig |
| RanBP2 | Santa Cruz Biotechnology | sc-74518 |
| p-G3BP1(Ser149) | Affinity Bioscience | AF8478 |
| p-Ser/Thr | ECM Bioscience | PP2551 |
| CSNK2A1 | Proteintech Group | 10992-1-AP |
| Phospho-EIF2S1 (Ser51) | Proteintech Group | 28740-1-AP |
| EIF2S1 | HUABIO Bioscience | HA500385 |
| MDM2 | HUABIO Bioscience | RT1382 |
| Alpha-Tubulin | Proteintech Group | 11224-1-AP |
| MYC-tag | Proteintech Group | 16286-1-AP |
| MYC-tag | Proteintech Group | 60003-2-Ig |
| HA-tag | Proteintech Group | 51064-2-AP |
| HA-tag | Proteintech Group | 66006-2-Ig |
| Flag-tag | Proteintech Group | 20543-1-AP |
| Flag-tag | Zen-Bioscience | 390002 |
| GFP | Cell Signaling Technology | #2956 |
| GFP | Zen-Bioscience | 300943 |

**Supplementary table 2 Sequences of siRNAs and shRNAs used in present study**

| Genes |  | Primer sequences (5’-3’) |
| --- | --- | --- |
| *TSPYL5* |  | Forward: TAATACGACTCACTATAGGG |
|  |  | Reverse: CTGGAATAGCTCAGAGGC |
| *GAPDH* |  | Forward: ACGGATTTGGTCGTATTGGG |
|  |  | Reverse: CGCTCCTGGAAGATGGTGAT |
| *SERPINE1* |  | Forward: AGTGGACTTTTCAGAGGTGGA |
|  |  | Reverse: GCCGTTGAAGTAGAGGGCATT |
| *DRAM1* |  | Forward: AGTGCTTGGATTGGTGGGATG |
|  |  | Reverse: GATGGACTGTAGGAGCGTGTA |
| *CDKN1A* |  | Forward: TGTCCGTCAGAACCCATGC |
|  |  | Reverse: AAAGTCGAAGTTCCATCGCTC |
| *RRM2B* |  | Forward: AGAGGCTCGCTGTTTCTATGG |
|  |  | Reverse: GCAAGGCCCAATCTGCTTTTT |
| *SHISA5* |  | Forward: ACCTGTGATGACCAATACTGCT |
|  |  | Reverse: TCCACCGGCTCTACACTGG |
| *BAX* |  | Forward: CCCGAGAGGTCTTTTTCCGAG |
|  |  | Reverse: CCAGCCCATGATGGTTCTGAT |
| *FAS* |  | Forward: T CTGGTTCTTACGTCTGTTGC |
|  |  | Reverse: CTGTGCAGTCCCTAGCTTTCC |
| *TP53* |  | Forward: ACTTGTCGCTCTTGAAGCTAC |
|  |  | Reverse: GATGCGGAGAATCTTTGGAACA |
| *G3BP1* |  | Forward: CGGGCGGGAATTTGTGAGA |
|  |  | Reverse: TCTGTCCGTAGACTGCATCTG |

**Supplementary table 3 Primers for RT-qPCR analysis**

| Definition | Sequences (5’-3’) |
| --- | --- |
| siNC | TTCTCCGAACGTGTCACGTAA |
| siG3BP1#1 | CATTAACAGTGGTGGGAAA |
| siG3BP1#2 | AGGCTTTGAGGAGATTCAT |
| siRanBP2#1 | GCTACAAGATGTGTTGCTT |
| siRanBP2#2 | AAGGACAGTGGGATTGTAGTG |
| siCSNK2A1 | GTCAGCAGCGCCAATATGA |
| SiTP53#1 | CGGCGCACAGAGGAAGAGAAT |
| SiTP53#2 | GTCCAGATGAAGCTCCCAGAA |
| shTSPYL5#1 | GCTTCTCATTCCACTCCAATT |

**Supplementary table 4 Top 50 DEGs obtained from the RNA-seq of the TSPYL5-knocked down SK-N-SH cells**

**Supplementary table 5 Top 50 DEGs obtained from the RNA-seq of the TSPYL5-knocked down SH-SY5Y cells**
