# Supplementary figures and images for: TSPYL5-driven G3BP1 nuclear membrane translocation facilitates p53 cytoplasm sequestration via accelerating RanBP2-mediated p53 sumoylation and nuclear export in neuroblastoma
Source: Cell Death Dis. 2025 May 3;16(1):358. doi: 10.1038/s41419-025-07694-x (PMC12049415; doi:10.1038/s41419-025-07694-x)

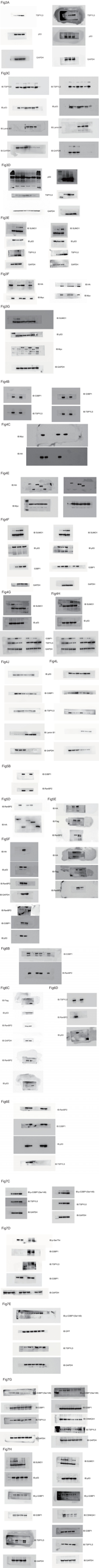

Supplement: Supplementary file 2 — Original data for Blots-Figure [file 41419_2025_7694_MOESM2_ESM.pdf]
